# Dynamic genome-scale cell-specific metabolic models reveal novel inter-cellular and intra-cellular metabolic communications during ovarian follicle development

**DOI:** 10.1186/s12859-019-2825-2

**Published:** 2019-06-10

**Authors:** Beatriz Peñalver Bernabé, Ines Thiele, Eugene Galdones, Anaar Siletz, Sriram Chandrasekaran, Teresa K. Woodruff, Linda J. Broadbelt, Lonnie D. Shea

**Affiliations:** 1 Department of Pediatrics, University of California San Diego, La Jolla, CA 92093 USA; 2 Luxembourg Center for Systems Biology, University of Luxembourg, Esch-sur-Alzette, Luxembourg, L-4365 Luxembourg; 3 Department of Obstetrics and Gynecology, Northwestern University Feinberg School of Medicine, Chicago, IL 60611 USA; 4 Department of Biomedical Engineering, University of Michigan, Ann Arbor, MI 48109 USA; 5 Women’s Health Research Institute, Northwestern University Feinberg School of Medicine, Chicago, IL 60611 USA; 6 Department of Chemical and Biological Engineering, Northwestern University Feinberg School of Medicine, Evanston, IL 60208 USA

**Keywords:** Ovarian follicle development, Metabolism, Metabolic communities, Secreted metabolites, Cell-type specific metabolic models, Genome-scale modeling

## Abstract

**Background:**

The maturation of the female germ cell, the oocyte, requires the synthesis and storing of all the necessary metabolites to support multiple divisions after fertilization. Oocyte maturation is only possible in the presence of surrounding, diverse, and changing layers of somatic cells. Our understanding of metabolic interactions between the oocyte and somatic cells has been limited due to dynamic nature of ovarian follicle development, thus warranting a systems approach.

**Results:**

Here, we developed a genome-scale metabolic model of the mouse ovarian follicle. This model was constructed using an updated mouse general metabolic model (Mouse Recon 2) and contains several key ovarian follicle development metabolic pathways. We used this model to characterize the changes in the metabolism of each follicular cell type (i.e., oocyte, granulosa cells, including cumulus and mural cells), during ovarian follicle development in vivo. Using this model, we predicted major metabolic pathways that are differentially active across multiple follicle stages. We identified a set of possible secreted and consumed metabolites that could potentially serve as biomarkers for monitoring follicle development, as well as metabolites for addition to in vitro culture media that support the growth and maturation of primordial follicles.

**Conclusions:**

Our systems approach to model follicle metabolism can guide future experimental studies to validate the model results and improve oocyte maturation approaches and support growth of primordial follicles in vitro.

**Electronic supplementary material:**

The online version of this article (10.1186/s12859-019-2825-2) contains supplementary material, which is available to authorized users.

## Background

Understanding the complex intercellular metabolic interactions during ovarian follicle development requires a systems biology approach. The follicle consists of somatic cells that surround the female germ cell, the oocyte. Metabolic communication between these cell types is necessary for follicle development and oocyte maturation. Yet, most systems-level studies of the follicle to date have focused on signaling and gene regulation [1] rather than on metabolism. While the metabolic interaction between granulosa cells and oocytes during development has been documented [2–5], a systems biology analysis provides a comprehensive perspective that is not possible using bottom-up methods measuring a few components at a time [6, 7]. Current untargeted metabolomics methods are not feasible for studying ovarian follicle metabolism due to the large number of cells (> 10,000) that are required, specifically for oocyte isolation. One way to overcome this limitation is by applying a systems biology approach to model ovarian follicle metabolism using transcriptomics data. Systems biology approaches can reveal key secreted and consumed metabolites, and dynamic metabolic processes that occur during mouse folliculogenesis in the oocyte and somatic cells.

Here we apply *genome-scale network models* to model follicle metabolism [8]. These network models are manually curated and represent the relationship between genes, proteins and metabolites in a system. They have been successfully employed to study of the metabolism of unicellular and multicellular organisms [9], including mammals [10]. The metabolic network models for multicellular organisms contain all possible biochemical reactions that happen in an organism based on literature evidence. For example, the human network model by Thiele et al. contains 7440 reactions, 1789 genes, 2194 transcripts, 2657 proteins, 1052 protein complexes, and 5063 metabolites [11]. Transcriptomics, proteomics or metabolomics data can be integrated with genome-scale metabolic models to create context-specific or cell-type specific models that represent metabolic reactions that are active in a cell-type. Such context-specific models have been applied successfully to predict metabolic behaviors of human and mouse tissues [12–15].

To build our cell-type specific metabolic models, we used the mouse metabolic reconstruction [16], and updated it based on the more comprehensive human metabolic model [11]. Using this updated mouse metabolic reconstruction and transcriptomic data of ovarian follicle cells, we next built a cell-type specific mouse ovarian follicle metabolic reconstruction [17]. We then explored this model to identify the most active metabolic communities and pathways. We further identified secreted and consumed metabolites at each stage of mouse ovarian follicle development for each cell type (e.g., oocyte, cumulus granulosa cells). Our study provides insights on the communication and dependence of the multiple cells types that comprise the ovarian follicle. Secreted and consumed metabolites identified by this approach in the growing ovarian follicle can be used to improve in vitro follicle culture systems, and to develop novel biomarkers of oocyte quality for in vitro fertilization (IVF).

## Results

### Updating the mouse general metabolic model

A comprehensive mouse metabolic reconstruction based on the most up-to-date metabolic knowledge could increase the accuracy of a reconstruction. Mouse Recon 1 was unable of adequately modelling multiple mouse metabolic functions, several of them associated with key follicle metabolic pathways (e.g., the production of estrogen metabolites). Thus, we constructed a high quality and more comprehensive mouse metabolic reconstruction, called Mouse Recon 2, employing the current best practices in systems biology [11] (Additional files 1 and 2). Mouse Recon 2 combines the previous established Mouse Recon 1 [16] with the metabolic pathways that have human homologues in the human metabolic reconstruction, Human Recon 2 [11] and several key ovarian follicle development metabolic pathways that were not included in either of the two reconstructions (Additional file 9: Note S1 and Note S2). The new Mouse Recon 2 contained a total of 2082 new reactions and 754 new unique metabolites (Table 1). Out of these new reactions, 700 of them were catalyzed by 251 enzymes that were not previously included in Mouse Recon 1. The genes that encode these new enzymes were highly enriched in oxidative phosphorylation processes and androstenedione and testosterone biosynthesis and metabolism (Additional files 8 and 9: Table S1).

**Table 1:** Comparisons between Mouse Recon 1 and Mouse Recon 2

Property	Recon 1	Recon 2
Total number of reactions	4091	6172
Total number of metabolites	2950	4380
Number of unique metabolites	1535	2293
Number of metabolites in extracellular space	469	567
Number of metabolites in cytoplasm	1029	1357
Number of metabolites in mitochondrion	393	732
Number of metabolites in nucleus	95	155
Number of metabolites in endoplasmic reticulum	238	505
Number of metabolites in peroxisome	143	422
Number of metabolites in lysosome	219	251
Number of metabolites in Golgi apparatus	281	305
Number of unique genes	1776	2123
Number of subsystems	102	114
Number of blocked reactions (% of all reactions)	789 (19%)	1496 (24%)^a^
Number of dead-end metabolites	510	1181^a^
Number of metabolic functions (out of 363)	304	338^a^

^a^Before gap analysis: 1572 blocked reactions, 1254 dead ends and carry flow 337 of the evaluated functions

Comparison of the metabolic pathways between Mouse Recon 2 and Mouse Recon 1 showed that 12 metabolic pathways were completely new, such as androgen and estrogen metabolism, arachidonic acid metabolism, and cytochrome metabolism. A total of 51 metabolic pathways were updated, some of these are known to be involved in ovarian follicle maturation, e.g., vitamin D, cholesterol, and steroid metabolism (Additional file 9: Figure S1). Additionally, we identified 43 genes in Mouse Recon 2 that have human homologues but were not included in Human Recon 2 (Additional file 9: Table S2). Finally, we checked the model metabolic functionality (Additional file 3). Out of 363 distinct metabolic functions (e.g., production of ATP from glucose), Mouse Recon 2 was able to successfully simulate 93% of the tested metabolic functions, while Mouse Recon 1 could only simulate 84% of those functions.

### Creation of a mouse ovarian follicle specific metabolic reconstruction

**Fig. 1:**
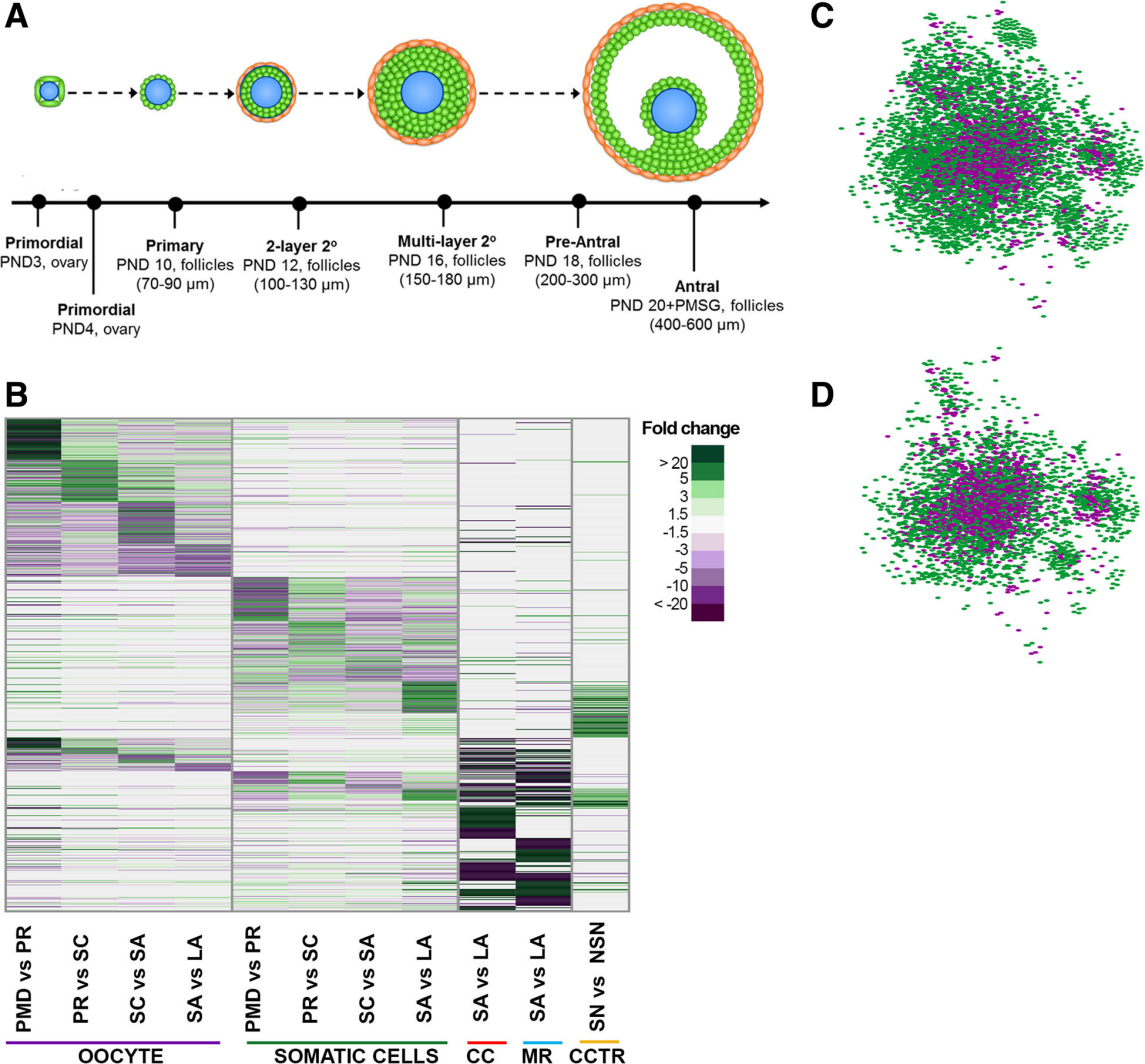
Reconstruction of the ovarian follicle metabolic model, OvoFol Recon 1, based on transcriptomic data. **a** Follicle sizes and age of the mice that the follicles were collected from; **b** Heatmap of the top significantly expressed genes for each follicle stage and follicle cell type; **c** Bi-partite graph of the Mice Recon 2 metabolic model, showing enzymes in purple and metabolites in green; **d** Bi-partite graph of OvoFol Recon 1 metabolic model constructed using FASTCORE from Mouse Recon 2. PND, post-natal day; PMSG, pregnant mare serum gonadotropin; PREANTRAL, pre-antral follicles between 200 and 300 μm; ANTRAL, antral follicles, between 400 and 600 μm; PMD, primordial; PR, primary; 2LS, two layered secondary; MLS, multi-layer secondary; SC, secondary follicle; SA, small antral; LA, large antral; CC, cumulus cells; MR, mural cells; CCTR, cumulus granulosa cells in large antral follicles during the acquisition of oocyte competence; NSN, non-surrounded oocyte nucleolus; SN, surrounded oocyte nucleolus

Among the 6172 reactions in Mouse Recon2, only a small subset of reactions is likely to be active in follicle cells. We hence constructed an ovarian follicle specific metabolic model, OvoFol Recon 1 (Methods; Additional files 4 and 5). OvoFol Recon 1 was obtained by integrating four sets of mouse follicles transcriptomics data (Additional file 9: Table S3). This transcriptomics set includes our data from freshly isolated follicles and oocytes [18] across all stages of follicle development, cumulus cells and mural cells during in vivo follicle maturation [19], cumulus cells during the in vivo acquisition of oocyte competence [20] (Fig. 1a, b). OvoFol Recon 1 contained 3992 reactions, 1364 unique metabolites, and 1871 genes (Table 1). OvoFol Recon 1 has 2180 reactions fewer than Mouse Recon 2 (Fig. 1d, e)**.** Out of the total 336 metabolic function tested, OvoFol Recon 1 successfully simulated 246 functions (Additional file 3). A total of 1212 follicular genes that encodes for enzymes were identified in the four transcriptomics datasets (Additional file 9: Table S3) and were also present in Mouse Recon 2, out of these 1212 follicular genes, 1078 were present in OvoFol Recon 1 too. The remaining follicular genes that encode for enzymes did not have any functional metabolic reaction associated with them and were therefore excluded from the reconstruction. This model provides a comprehensive map of the mouse ovarian follicle metabolism that can be mined to identify active metabolic pathways in the female germ cell line and the metabolites that it consumes or secretes.

### Network analysis of the mouse ovarian follicle metabolic reconstruction

**Fig. 2:**
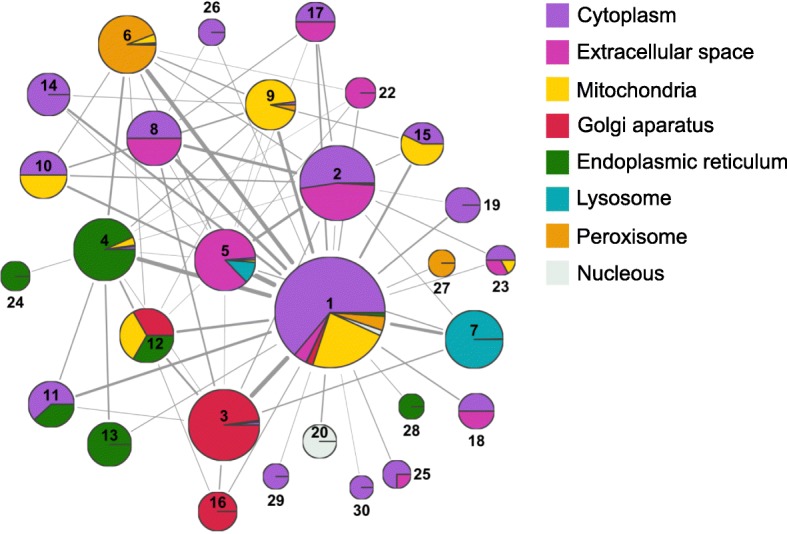
Ovarian follicle metabolic communities in OvoFol Recon 1 based on metabolite flow between enzymes (from Infomap). The sizes of the communities, defined as clusters of highly connected enzymes based on the flow of information through them, and the width of the edges between communities are proportional to the information that flows through them. Communities are color-coded based on the proportion of enzymes that pertain to a given location. For instance, Community 1 is largely composed of cytoplasmic enzymes (purple), followed by mitochondrial enzymes (yellow)

Next, we established the major metabolic pathways that were differentially active across multiple follicle stages using a network approach. Superimposing transcriptomic information in enzyme ovarian follicle metabolic network is a powerful approach to identify active metabolic pathways. [21]. We created an enzyme ovarian follicle metabolic network by connecting enzymes that share common metabolites. Highly inter-connected enzymes within a network are called communities. Communities are clusters of members (e.g., enzymes, genes) that have more connections between themselves than with other members in the network. OvolFol Recon 1 was divided into 30 communities (Additional file 6), or clusters of highly interconnected enzymes (Fig. 2), according to Infomap [22, 23], one of the leading community detection methods [24]. Infomap decomposes a network into communities based on what is called information flow through the network. Information flow between enzymes that are closely connected, i.e., they share common metabolites, is greater than between enzymes that do not possess any common metabolites. Hence, communities are formed by enzymes that support high levels of information flow between them.

Subsequently, we overlaid transcriptional data for each ovarian follicle cell type into OvolFol Recon 1. Cell-type specific genes that encode for enzymes were overlapped over OvolFol Recon1 to render oocyte-, somatic-, cumulus- and mural-specific metabolic models. To determine cell-type specific metabolic pathways, we calculated a normalized flow through each community. This normalized flow through each community was quantified using Eq. 1 (Methods), and accounts for differences in community sizes and connection between them; it further accounts for changes in abundance of transcripts encoding enzymes that participate in each community and the number of metabolites that each enzyme catalyzes. Thus, the most transcriptionally-active and highly connected communities in the network will have the largest normalized flow.

Normalized flow revealed metabolic patterns for the multiple cell types and follicle stages during ovarian follicle development (Additional file 9: Figure S3). Notably, the metabolic communities in primordial oocytes differed significantly from that of oocytes at other follicle stages (Additional file 9: Figure S3). Peroxisomal (Community 6) and lysosomal (Community 7) processes were more prominent in the oocyte at the early stages of follicle development, while mitochondrial processes in the oocyte were enhanced over time (Community 9). The enhancement of oocyte mitochondrial processes might be associated with the observations that oxidative phosphorylation is more prominent at the later stages during follicle development once that the antrum is formed [7], as oxygen is more accessible for the oocyte, probably from the follicular fluid [25, 26]. Compared to the oocyte, the somatic cells (granulosa and theca cells) had greater metabolic activity in the cytosol (Community 1) and the endoplasmic reticulum (Communities 4, 12, and 22), with the latter especially active during the antral stage. When comparing cumulus cells and mural cells during antral formation, cumulus cells showed more metabolic processes activated in the lysosomes (Community 7), whereas mural cells had more activity in the peroxisome (Community 6). Interestingly, during the acquisition of oocyte competence, characterized by the transition from non-surrounded oocyte nucleolus (NSN) to the surrounded oocyte nucleolus (SN), cumulus cells present in large antral follicles during the transition exhibited more prominent peroxisomal processes (Community 6) than lysosomal processes (Community 7). Note that mitochondrial processes were mostly silent in cumulus cells (Community 9) and were only active in the incipient mural cells**.**

### Enriched metabolic pathways during follicle development

We next identified metabolic pathways, as defined by the Kyoto Encyclopedia of Genes and Genomes [27], that are over-represented in the cell-type specific metabolic communities. Pathway enrichment was calculated as the normalized flow of all the genes that belong to a specific pathway (e.g., pyruvate metabolism) compared with the background normalized flow of the equal number of randomly selected genes for the entire network (Methods).

**Fig. 3:**
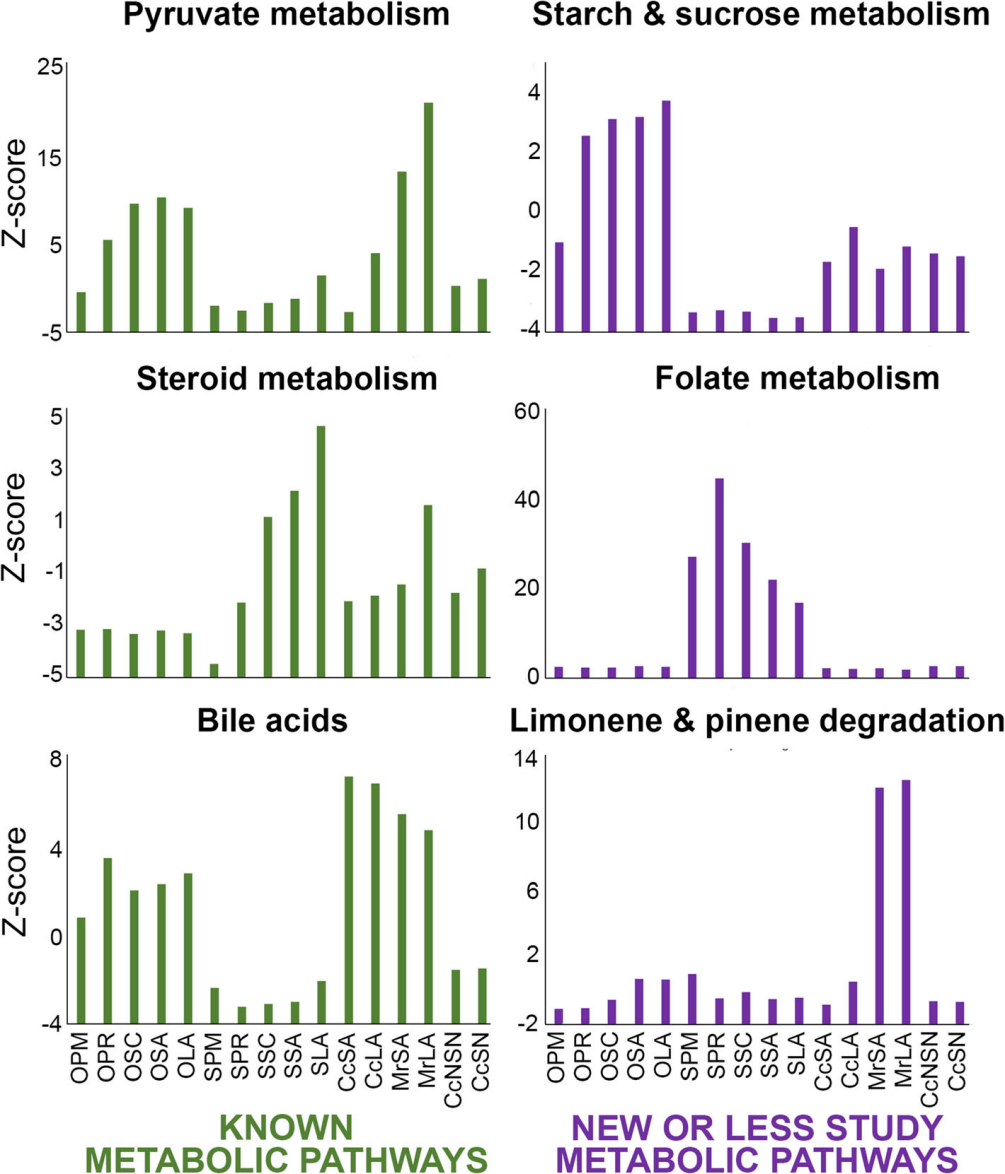
Top metabolic pathways in each follicle cell type based on Z-scores, which accounts for the transcriptional activity of the genes that encode the corresponding enzymes in the metabolic pathway and the flow of information between those enzymes, during ovarian follicle maturation. Metabolic pathways are divided based on the abundance of scientific references during ovarian follicle development. OPM, oocyte primordial; OPR, oocyte primary; OSC, oocyte secondary; OSA, oocyte small antral; OLA, oocyte large antral; SPM, somatic primordial; SPR, somatic primary; SSC, somatic secondary; SSA, somatic small antral; SLA, somatic large antral; CcSA, cumulus small antral; CcLA, cumulus large antral; MrSA, mural small antral; MrLA, mural large antral; CcNSN, cumulus granulosa cell in large antral follicles that present a non-surrounded oocyte nucleolus; CcSN, cumulus granulosa cell in large antral follicles that present a surrounded oocyte nucleolus

Analysis of information flows based on the connectivity of the metabolic network and the transcriptomic data allowed identification of the most enriched metabolic pathways in the cell-specific oocyte and somatic cell metabolic models during follicle development (Fig. 3). The enrichment analysis identified well-known metabolic pathways that occur during follicle development, such as pyruvate metabolism in the oocyte [26], the production of estrogen in granulosa cells in antral follicles [26], and the production of bile acids [28]. Moreover, we were also able to pinpoint new or less studied pathways, such as folate acid metabolism in somatic cells, starch and sucrose metabolism in the oocyte, and limonene and pinene degradation in the mural cells.

Most of the enriched metabolic pathways belonged to Communities 1 and 2, according to the most active and differentiated pathways among diverse cell types (Additional file 7). At the primordial stage, the enriched metabolic pathways in the oocyte were arginine and proline metabolism and oxidative phosphorylation, whereas folate metabolism and fatty acid oxidation were among the most active metabolic pathways in primordial somatic cells –note that in this case somatic cells includes squamous granulosa cells and stroma cells that surround the oocyte. Fatty acid oxidation was active in the primordial follicle, both in the oocyte and in the somatic cells, providing a source of energy for the early follicle. This observation is consistent with previous studies that have suggested that glycogen is one of the sources of energy in primordial germ cells [26]. In contrast, the top significant metabolic pathways in oocytes in primary, secondary, and antral follicles were coenzyme catabolism and fatty acid oxidation in the peroxisome. Somatic cells of primary, secondary, and antral follicles had highly active metabolism of folic acid and nucleotides, extracellular transport (Community 2), and heme production.

During the formation of the antrum and the differentiation of granulosa cells into cumulus and mural cells, the ranking of metabolic pathways in terms of their Z-scores changed with respect to the other cell types. Inosinic acid and carnitine shuttling were among the top metabolic pathways in cumulus cells; in contrast, mural cells showed highly active pyruvate metabolism, limonene and pinene degradation, o-glycan synthesis, and transport to the lysosome. Finally, during the acquisition of oocyte competence, the most active pathways in cumulus cells were fatty acid oxidation and cholesterol and propionate metabolism.

### Key metabolites during mouse ovarian follicle maturation

**Fig. 4:**
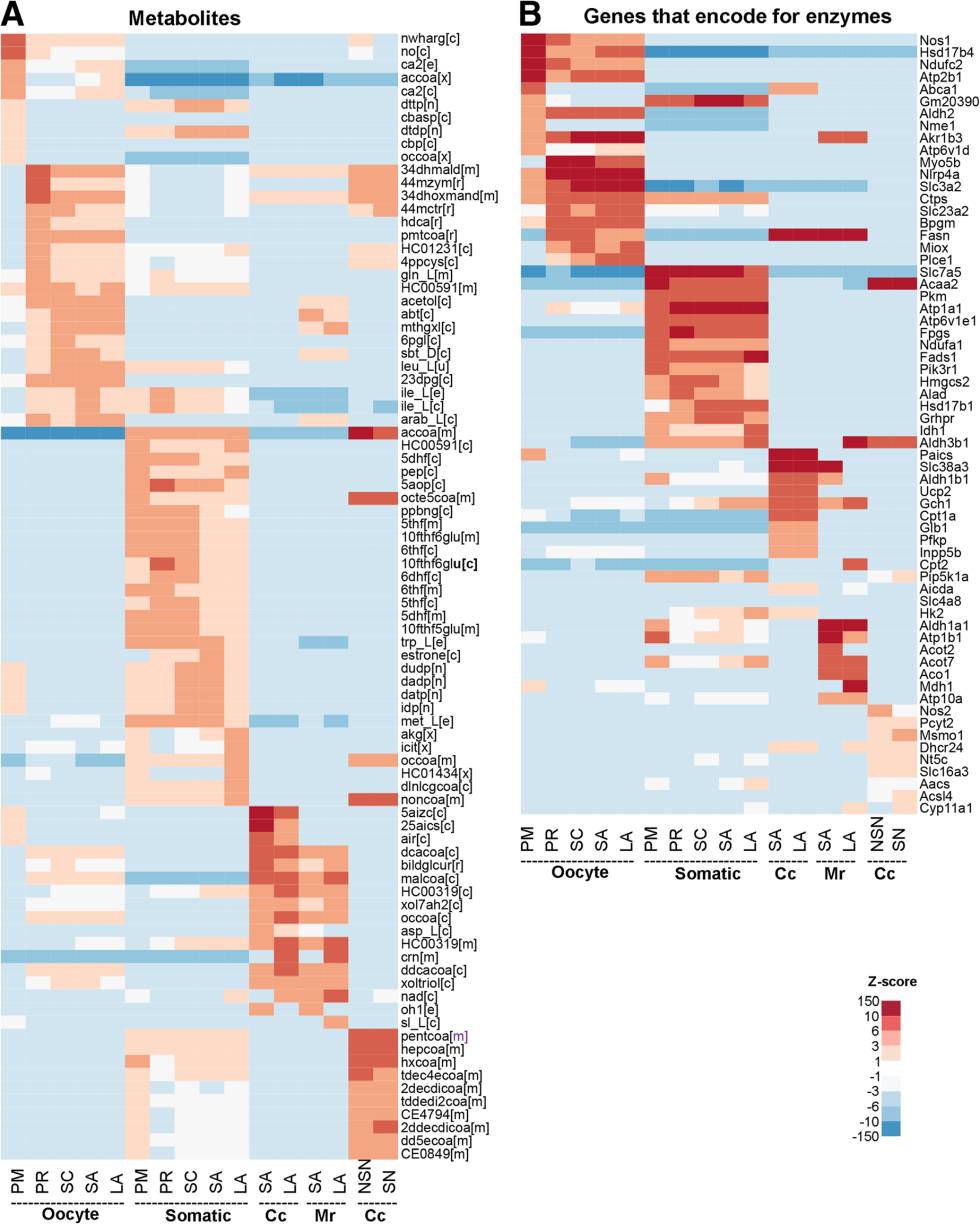
Top metabolites (**a**) and genes (**b**) encoding enzymes in each follicle cell type during follicle development. Cc, cumulus cells; Mr., mural cells; PM, primordial; PR, primary; SC, secondary; SA small antral; LA, large antral follicle. Metabolite and enzyme full names can be found in Additional file 2

We next identified the most significant metabolites using a similar approach to the identification of over-represented pathways. Key metabolites were uncovered by measuring the total information flow through the enzymes that catalyze the reactions in which the metabolite participates (Methods). We identified the top 10 metabolites for each follicle stage and cell type (Fig. 4a). Top metabolites for oocytes in primordial follicles did not align with those in oocytes in other follicle stages. For instance, nitric oxide (NO) in the cytosol and the intracellular cytosolic and extracellular calcium ion (Ca^2+^) were the most important metabolites in primordial oocytes, while 6-phospho-D-glucono-1,5-lactone and 2,3-bisphosphonato-D-glycerate, which are involved in the pentose phosphate pathway and oxygen release from red blood cells, respectively, were more prominent in oocytes at later follicle stages (Fig. 4a).

Interestingly, significant metabolites in the somatic cells were similar at all follicles stages and were primarily folic acid derivatives and L-methionine, which are involved in DNA methylation. L-tryptophan had a high enrichment Z-score in somatic cells as well. This amino acid is the precursor of serotine, which has known effects in follicle maturation [29]. Cytosolic estrone did not become a significant metabolite in the somatic cells until the secondary stage and onwards, as expected. Cumulus granulosa cells during the small to large antral transition showed a significant activation of metabolites related to purine metabolism (e.g., 5aizc, 25aics, air). Lipids are known to play an important role during the acquisition of oocyte competence [30], and were indeed overrepresented in the cumulus cells during the NSN to SN transition in the oocyte to acquire its competence (e.g., pentanoyl-coa, heptanoyl-coa, hexanoyl-coa).

### Key genes that encode for enzymes during follicle maturation

Similarly, we identified the top 10 genes encoding enzymes in each cell type and follicle stage based on their Z-scores (Fig. 4b). The most significantly expressed genes encoding enzymes in the oocytes of primordial follicles differed from those expressed in oocytes at other follicle stages. *Nos1* (nitric oxide synthase) and *Hsd17b4* (hydroxysteroid 17-beta dehydrogenase 4), which is an enzyme part of the peroxisomal beta-oxidation pathway for fatty acids, were the two top enzymes in primordial oocytes; whereas *Myo5b* (Myosin Vb), an effector for RAB11A required for recycling of transferrin in nonpolarized cells [31], *Akr1b3* (aldo-keto reductase family 1, member B3), which participates in pyruvate metabolism, and *Scl3a2*, a glutamine transporter, were among the most enriched genes encoding enzymes in oocytes of all other follicle stages (Fig. 4b).

In the somatic cells, the top most significant genes encoding enzymes based were *Slc7a5*, *Atp1a1* (ATPase Na+/K+ transporting subunit alpha 1), *Fpgs* (folylpolyglutamate synthase), and *Fdas1* (fatty acid desaturase 1). *Slc7a5* encodes an amino acid transporter involved in high-affinity transport of large neutral amino acids such as phenylalanine, tyrosine, leucine, arginine, and tryptophan, while *Fpgs* encodes an enzyme that establishes and maintains both cytosolic and mitochondrial folylpolyglutamate concentrations and, therefore, is essential for folate homeostasis and the survival of proliferating cells. The enzyme encoding by *Fpgs* catalyzes the conversion of folates to polyglutamate derivatives allowing to maintain the concentrations of folate components in the cell. *Fpgs* also facilitates the intracellular retention of these cofactors, which are important substrates for most of the folate-dependent enzymes that are involved in one-carbon transfer reactions in purine, pyrimidine, and amino acid synthesis. *Fdas1 Isoform 1*, which has its highest Z-score values in the large antral follicles, encodes a component of a lipid metabolic pathway that catalyzes the biosynthesis of highly unsaturated fatty acids and generates arachidonic acid. Arachidonic acid increases the concentration of estrogen and progesterone in granulosa and theca cells [32]. *Hsd17b1* (hydroxysteroid 17-beta dehydrogenase 1) encodes an enzyme involved in the metabolism of estrogens, and reduces both estrogens and androgens (Fig. 4b). Highly ranked genes in cumulus cells were *Paics*, which is involved in purine biosynthesis, and *Aldh1a1* (aldehyde dehydrogenase 1 family member A1) in mural cells, a gene that encodes an enzyme that produces retinoic acid, an important vitamin component in ovarian follicle development [33].

### Exo- and endo-metabolites during in vivo follicle maturation

**Fig. 5:**
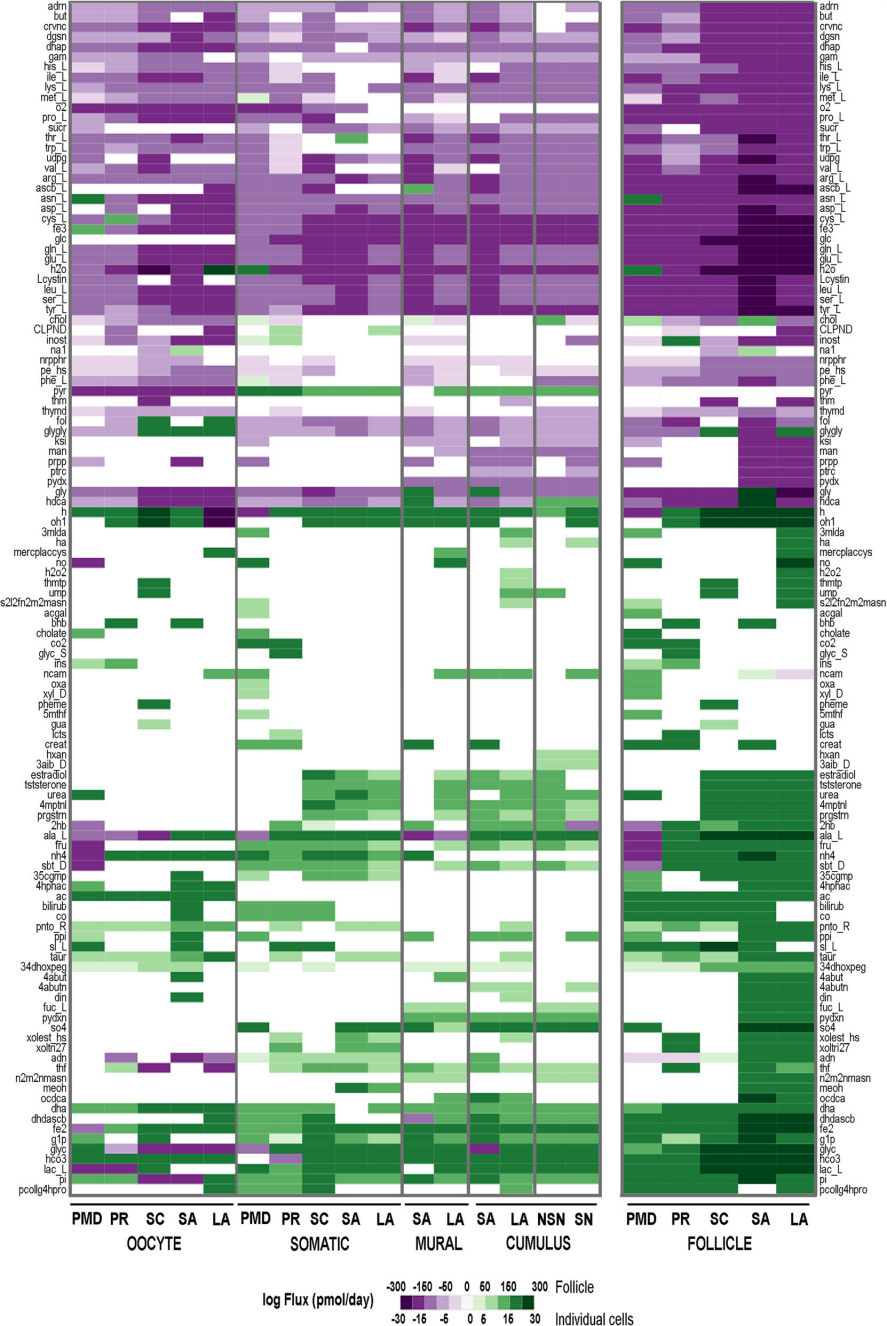
Predicted exo- and endo-metabolism in each follicle cell type during follicle maturation. PMD, primordial follicle; PR, primary; SC, secondary; SA, small antral follicle; LA, large antral follicle; NSN, non-surrounded oocyte nucleolus; SN, surrounded oocyte nucleolus. Extracellular reactions and metabolite full names can be found in Additional file 2

Finally, we determined the most likely secreted and consumed metabolites by each cell type at each follicle stage (Fig. 5). We simulated each stage- and cell-type specific metabolic models, using experimentally measured metabolites that are consumed or produced during follicle development (e.g., glucose, oxygen, Additional file 9: Table S4) to constrain the metabolic models. The plasma composition of these metabolites was employed for these calculations (Additional file 9: Table S5). Our computational results were consistent with several reports on multiple metabolites: i) the consumption of nitric oxide by the oocyte, which prevent apoptosis [34]; ii) consumption of fructose, sorbitol, and L-lactose [26] by the oocyte, which somatic cells produce; iii) L-alanine uptake by oocytes and secretion of L-alanine at later stages [35]; iv) hypoxanthine production in cumulus cells during the time of the oocyte is acquiring competence to inhibit oocyte maturation [36, 37], as well as the production of L-fucose [38]; and iii) collagen production only by the somatic cells that requires ascorbic acid, which has been recently demonstrated by our group [39]. Ascorbic acid allows the survival of smaller follicles by supporting the production of more extracellular matrix (ECM) components [39]. Supplementation of the α-MEM media that is currently used in in vitro follicle culture has shed some light into the long-standing challenge of growing primary follicles in 3-D alginate gels [39].

Our analysis also discovered novel metabolic processes during ovarian follicle maturation. For example, our model suggests that the oocyte produces metabolites acetate and inosine. Acetate is the source of cholesterol for cumulus cells [40], and based on our computational results, the origin of that acetate is the oocyte itself. Inosine maintains the meiotic arrest of oocytes [36], but it is also produced by the oocytes themselves, and not by the cumulus cells, as it happens with hypoxanthine. Somatic cells, from primordial follicles all the way to antral follicles, consumed folic acid. Additionally, the oocyte produced folic acid, specifically at the later stages of ovarian follicle development. Another similar component, vitamin B6 (pyridoxine) was produced by the cumulus and mural cells. At the follicle level, the model predicted that most of the amino acids were consumed except for the non-essential amino acid taurine, and sucrose.

## Discussion

In this study, we created and analyzed the first metabolic model of the mouse ovarian follicle and its cellular compartments—the oocyte and somatic cells (i.e., granulosa and theca cells)—through the stages of follicle development. We used a systems biology approach to decipher the key exo- and endo-metabolic processes present during mouse ovarian folliculogenesis in vivo. For this purpose, the latest mouse metabolic reconstruction, Mouse Recon 1, was updated based on the recently developed human reconstruction, Human Recon 2. This new model was able to successfully simulate an additional 10% of the metabolic functions compared to the Mouse Recon 1 model. Mouse Recon 2 was then employed to generate the first mouse ovarian follicle metabolic reconstruction, OvoFol Recon 1. Mouse Recon 2 could be applied in future studies to predict mouse phenotypes using IMPC [41] (http://www.mousephenotype.org/). Similarly, OvolFol Recon 1 could seed light into human diseases associated with ovarian follicle development (e.g., https://rarediseases.info.nih.gov/diseases/diseases-by-category/10/female-reproductive-diseases).

Our new method based on the combination of network approaches and transcriptional activity identified the most relevant metabolic pathways, metabolites and metabolic genes. We were able to identify cell-specific metabolic pathways that occur during follicle development (e.g., pyruvate metabolism in the oocyte [26], the production of estrogen in granulosa cells in antral follicles [26] and the production of bile acids [28]). Our approach also identified new or less studied pathways, such as folate acid metabolism in somatic cells, starch and sucrose metabolism in the oocyte, and limonene and pinene degradation in the mural cells.

Our new methods allowed the exploration of the variation in metabolites and enzymes and may support future studies on the communication between the multiple cellular compartments within the follicle and the metabolic changes within other multi-cellular systems. For instance, we were able to reveal two interesting examples in ovarian follicle metabolism: folic acid and taurine. Folate metabolism has been extensively studied due to its implications on fetal outcomes, as it alters the DNA methylation profiles of the oocyte together with methionine [42], but the origins and effects of folate during follicle formation are relatively unknown. In fact, our model suggests the follicle consumes folic acid. Taurine has been measured before in the mouse follicular fluid (Additional file 9: Table S5), and it was speculated that the large concentration of taurine in the follicular fluid was due to its accumulation, as it was not consumed by the follicle [6]. Based on our model, taurine is produced by the oocyte and the somatic cells beginning from the primary stage, and even in the primordial somatic cells. Taurine has several physiological actions [43] – it is a potent anti-oxidant [44] and intervenes in calcium transport [45]. At this point, the effects of taurine in ovarian follicle maturation are not clear; however, it is tempting to hypothesize that taurine is produced by the oocyte and somatic cells to protect the oocyte against radical oxidant species and improve calcium transport required for downstream signaling of follicle-stimulating hormone (FSH).

Identification of the metabolites that are secreted or consumed by the ovarian follicle during its development has two-fold significance: a) secreted metabolites may be employed as biomarkers for the stage of follicle development, which is necessary to monitor the growth in vitro of follicles from large mammalian species whose stage cannot be monitored under the microscope. Several metabolites may be useful for determining follicular stage. The transition from primordial follicles to primary follicles could be detected by the decrease of production of nitric oxide, oxalic acid, D-xylose, cholate, or 5-methyltetrahydrofolate. Similarly, the primary to secondary transition could be monitored based on the decrease in production of inosine or the production of (R)-3-hydroxybutyrate; b) consumed metabolites can guide the development of novel media components that will support follicle growth, particularly in early-stage follicles, and in vitro maturation (IVM). For instance, based on the model predictions, supplementation of the α-MEM media that is currently used in in vitro follicle culture with sorbitol and 2-hydroxybutyrate or further increase the concentration of folic acid in the media, as the three metabolites are consumed by the follicles.

The metabolome of the oocyte differed greatly from that of the somatic cells at every stage of follicle development. This result is consistent with current thinking regarding the geography of the ovary, where primordial follicles, which are located in the ovarian cortex, and secondary and antral follicles, located in the medulla [46], are exposed to different metabolites. The limited success in growing primordial follicles in vitro without using two-step cultures [47] may be related to the distinct metabolic differences between early and later-stage follicles. In fact, current in vitro follicle culture approaches were developed based on later-stage follicles where a relatively large body of knowledge of their biology is available [26]. These media are being ineffectively applied to designed primordial follicle growth media and conditions. Tuning the media composition as the ovarian follicle grows and the oocyte matures in culture may ultimately enhance oocyte quality.

The aim of our study was to develop novel data-driven hypothesis that could serve as new areas of research to understand the complex and dynamic intra- and inter-cellular communication between the different ovarian follicular cell types. Our models can be improved by using experimental isolated cell types from all the stages during follicle development. Currently, there is no such a set in mice, especially for granulosa cells, most likely due to the technical difficulties. Similarly, it should be noted that the results from our model have been obtained using transcriptional data from pre-pubertal mice and those differ from pubertal mice, as the dynamics of ovarian follicle development is faster in younger mice and slows with mouse age [48]. Finally, predictions obtained from transcriptomic data alone does not guarantee the activity of a given enzyme. Herein, mRNA levels were employed as a proxy for enzymatic activity, while metabolomics and proteomics would be ideal [12, 13]. Established non-target metabolomic techniques typically require millions of cells [49], and even emerging techniques require on the order of thousands of cells [50], which is prohibited for oocyte isolation. Proteomics approaches that account for post-translational modifications would be desirable, however, non-proteomics data is only available at later ovarian folliculogenesis stages for entire follicles grown in vitro [51]. In fact, the combination of proteomics with the community network approach reported herein would have the potential to reveal the most important systems and key metabolites and enzymes, as it has been done before in human tissues [52].

## Conclusion

In conclusion, we successfully applied a systems biology approach to characterize the most important metabolic pathways in the oocyte and somatic cells during various stages of ovarian follicle development. The understanding of the follicle metabolome has been limited by decades of research using bottom-up approaches, which has provided only snapshots of the complex metabolic landscape of the growing follicle. Collectively, metabolic systems approaches were able to model the follicle metabolome, providing a rich set of data that can be applied to generate new hypothesis to test experimentally. Our study can improve in vitro follicle growth and oocyte maturation approaches and support the growth of primordial follicles in vitro.

## Methods

### Updating the mouse general metabolic model

We developed Mouse Recon 2, a general metabolic model based on the latest general human metabolic reconstruction, Human Recon 2.03 [53], employing a similar methodology to that applied for the first mouse metabolic reconstruction, Mouse Recon 1 [54]. The reconstruction was performed through a series of iterative steps aiming to reduce ambiguities when merging the two reconstructions, Mouse Recon 1 and Human Recon 2.03 (Additional file 8: Note S1) and was followed by a gap analysis evaluation using fastGapFill [55] (Additional file 8: Note S2). Mouse Recon 2 was examined against a total of 363 metabolic functions, such as production of biomass, production of pyruvate and lactate under anaerobic conditions from glucose, pyruvate consumption, hormone production (estrogen, androgen, testosterone, and progesterone), and fructose and sorbitol production (Additional file 3). Flux variance analysis was subsequently performed to determine the dead-end metabolites and blocked reactions (i.e., reactions that either their reactants are not produced by any other reactions or obtained from the cell media, or their metabolites are not consumed neither exported outside the cell). The resulting MATLAB Mice Recon 2 model is provided in Additional file 1, and the model reactions, metabolites, and genes are summarized in Additional file 2.

### Follicle collection and isolation

We followed procedures for ovary and follicle isolation as previously established [56] with slight modifications. CD-1 mice were obtained commercially from Harlan Laboratories, USA. CD-1 mice were housed in a temperature- and light-controlled environment (14 h light, 12 h dark) and provided with food and water ad libitum. Animals were fed Teklad Global irradiated 2919 low-phytoestrogen chow. At the time of delivery, 8 female pups were housed with each dam to minimize differences in pup development caused by nutrient availability. Animals were treated in accordance with the NIH Guide for the Care and Use of Laboratory Animals and the established IACUC protocol at Northwestern University. Donor mice were euthanized by CO2 inhalation followed by cervical dislocation. Entire ovaries were collected at post-natal day 3 and day 4 to collect primordial follicles. Primary follicles (70–90 μm in diameter), two-layered secondary follicles (100–130 μm), multi-layer secondary follicles (150–180 μm), and pre-antral follicles (200–300 μm) were mechanically isolated from post-natal day 10, 12, 16, and 18 ovaries, respectively. Antral follicles (400–600 μm) were mechanically isolated from pregnant mare serum gonadotropin (PMSG)-treated mice ovaries at post-natal day 20. Follicles were then aspirated and combined per ovarian follicle maturation stage (e.g., primary, two-layered secondary). Three different samples were collected from each pooled follicular stage for transcriptomic analysis. RNA was purified and hybridized in MouseRef-8 v2.0 Expression BeadChip Kit (Illumina, San Diego, CA), as previously described [57].

### Follicle transcriptome data

Microarray data were downloaded from Gene Expression Omnibus (GEO) using the GEOquery [58] and Array Express packages [59] from Bioconductor (http://www.bioconductor.org) for: a) mouse oocytes from primordial to large antral follicles [18] (E-GEOD-3351); b) mouse cumulus and mural cells collected during antrum formation (secondary to antral transition) [19] (GSE55845), and c) mouse cumulus cells collected at the time of oocyte competence acquisition (large antral follicles) [20] (E-GEOD-36617). The three published microarray datasets and our microarray data from the isolated mouse primordial to large antral follicles, as described above, were normalized and transformed and non-detected probes were removed as indicated in Additional file 9: Table S3 [60]. Significant genes were identified using *limma* [61] and were corrected for multiple comparisons using the false discovery rate (fdr) method [62]. Our microarray data are published as GSE97902.

### mRNA segregation process

Genes present in both oocyte and the follicle transcriptome were removed from the follicle transcriptome before statistical analysis if they met the two following conditions: a) they were significant in the oocyte microarray (FC ≥2.5 and fdr-corrected *p*-value ≤0.01); b) and their fold change in the follicle microarray was below 1.05 from for the primary to two-layered secondary transition- the oocyte transcripts are diluted due to the granulosa cell proliferation. The genes that satisfied these conditions were considered to be only expressed in the oocyte during ovarian follicle development. Genes in the follicle microarrays that were significant in the oocyte and in the follicle microarray but did not follow the patterns described in conditions b and c were classified as present in both cell types, i.e., oocyte and somatic cells (Additional file 8). The rest were classified as genes that are only transcribed in the somatic cells and their experimentally determined transcription abundance was employed in all the later calculations.

Genes from each microarray were classified as oocyte only, somatic only (granulosa and theca cells), cumulus cell only, cumulus cell during acquisition of oocyte competence only, or mural cell only if they were present only in their corresponding arrays and were not significant in any other microarray for the given significance cut-offs (Additional file 9: Table S3). Those genes that did not satisfy the above conditions were considered to be present in multiple cell types (e.g., if a gene A was significant in the oocyte and cumulus cells).

### Follicle metabolic reconstruction and analysis

To generate the mouse ovarian follicle metabolic reconstruction, which we named OvoFol Recon 1, we used ovarian follicle transcriptomic data (Additional file 9: Table S3) and the FASTCORE algorithm [17]. First, the relevant genes included in Mouse Recon 2 were identified by removing all the blocked reactions using the *fastcc* function from FASTCORE. Then, a consistent cell-specific mouse ovarian follicle reconstruction was established with the *fastcore* function. OvoFol Recon 1 was validated against 363 metabolic functions using plasma composition (Additional file 9: Table S5).

An enzyme-metabolite bi-partite graph and an enzyme network graph were constructed based on the resulting OvoFol Recon 1. The enzyme-metabolite bi-partite graph included connections between enzymes and the corresponding metabolites they catalyze. The enzyme network graph contained only edges between the enzymes that catalyze the same metabolite. The number of communities, defined as clusters or partitions of highly interconnected enzymes, in the OvoFol Recon 1 enzyme network graph was established with Infomap [22, 24] using 1000 iterations. Infomap decomposes a network into communities based on a description of information flows in the network. Information flow between enzymes that are closely related, i.e., based on common metabolites, is greater than between enzymes that do not possess any common metabolites. Hence, communities are formed by enzymes that support high levels of information flow between them. The normalized flow through each community was calculated using Eq. 1, which accounts for differences in community sizes as well as the dynamic changes in mRNA abundance of the genes encoding enzymes that participate in each community. The normalized flow through the community was calculated as follows:

1 $$ {f}_{N_i}=\frac{\sum \limits_{k=1}^{k={n}_i}{w}_k{I}_k{f}_k}{\sqrt{\sum \limits_{k=1}^{k={n}_i}{w}_k}} $$

where *f*_*Ni*_ is the normalized intensity flow of the community (or pathway, or metabolite) *i*, *f*_*k*_ is the flow calculated with Infomap for element *k* in the community *i*, *w*_*k*_ is the number of metabolites that are catalyzed by the enzyme *k* according to the enzyme-metabolite bi-partite graph, *I*_*k*_ is 1 if no microarray data was employed (Fig. 2), or the intensity value for the given gene in the transcriptomic data (Additional file 9: Figure S3). This normalized flow accounts for differences in community sizes as well as the dynamic changes in mRNA abundance of the genes encoding enzymes that participate in each community. By weighting the number of metabolites that each enzyme catalyzes, we accounted for the impact that each enzyme has at the metabolic level.

### Metabolic hot spots: communities, pathways, genes and metabolites

The most significant communities or “hotspots” in OvoFol Recon 1 were uncovered by measuring the total flow per community, normalized by the community size (Eq. 1). Z-scores for the metabolic pathways, metabolites, and genes were obtained by calculating the corresponding mean, *μ*, and standard deviation, *σ*, of 1000 randomly samples of the same size (Eq. 2). The values of the samples corresponded to randomly shifting the normalized intensity flows among all the elements without replacement.

2 $$ Z-{score}_i=\frac{{\mathrm{f}}_p-\mu }{\sigma } $$

Pathway enrichment within a community, *f*_*p*_, was calculated as the normalized intensity flow of all the genes or nodes in a given community that belong to a specific pathway within OvoFol Recon 1 (e.g., pyruvate metabolism). *f*_*p*_ was compared with the background normalized intensity flow of the equal number of randomly selected genes for the entire network using the enzyme-metabolite bi-partite graph. Enzyme Z-scores were established using Eq. 1, and *f*_*p*_ was determined based on the flow of the enzyme accounting for all the metabolites that are catalyzed by the given enzyme in the entire network, *w*_*k*_. Similarly, Z-scores of the metabolites were established using Eq. 1 and *f*_*p*_ was determined based on the flow of the enzymes that catalyze the reactions in which the metabolite participates, setting *w*_*k*_ to 1.

### Number of granulosa, theca, and cumulus cells during ovarian follicle development in vivo

Deparaffinized histological sections of the ovaries employed for the in vivo transcriptomic studies were used to determine the number of different cell types over time in each follicle stage (Additional file 9: Figure S2). The total number of granulosa cells, *n*_*G*_, was calculated as follows [63] (Eqs. 3 and 4):

3 $$ {n}_G=\frac{4}{3}{\pi \rho}_G\left({r}_{FwoT}^3-{r}_O^3-{r}_A^3\right) $$

4 $$ {r}_A=\sqrt{\frac{A_A}{\pi }} $$

where *ρ*_*G*_ is the granulosa cell density; *r*_*FwoF*_ is the radius radio of the follicle excluding the theca layer if present; *r*_*O*_ is the oocyte radius; *r*_*A*_ is the antral radius that was estimated assuming that the observed antral area; and *A*_*A*_, when present, was a sphere. The granulosa cell density was based on the observed granulosa volume in the image, taking into account that the slides were 5 μm thick, *f*, by the total number of manually counted granulosa cells in the slide (Eq. 5).

5 $$ {\rho}_G=\frac{f\pi \left({r}_{Fwo\mathrm{T}}^2-{r}_O^2-{A}_A\right)}{n_{Ginslide}} $$

The total number of theca and cumulus cells were estimated using the same approach. The cell type ratios were obtained from slides in which the nucleus of the oocyte could be observed. The average of the maximal and minimal axis of the follicle was employed as an estimation of the cellular ratios. All images were processed with ImageJ (Rasband, W.S., ImageJ, U. S. National Institutes of Health, Bethesda, Maryland, USA, http://imagej.nih.gov/ij/, 1997–2014).

### Cell-specific models and follicle exo- and endo-metabolism analysis

Metabolic networks within and between the oocyte and somatic cells during each stage of ovarian follicle development (primordial, primary, secondary, antral, and non-surrounding and surrounding nucleolus) were reconstructed using the FASTCORE algorithm [17] in the same manner as OvoFol Recon 1. For each of these follicle stage-specific reconstructions, the secreted metabolites were obtained (Additional file 9: Supplementary Note 3) and constrained with experimental parameters when they were available (Additional file 9: Table S4). Previously experimentally measured oxygen, glucose, and lactose consumption or production, hormone production, and hyaluronic acid production were collected from various sources in the literature (Additional file 9: Table S4). The plasma composition of metabolites was employed for these calculations (Additional file 9: Table S5). Total metabolite flow uptake was determined iteratively, so that the final estimated pyruvate flux coincided with the experimentally measured pyruvate production by the somatic cells. Metabolites produced or consumed by the somatic cells were added to or subtracted from the initial flux to the oocyte. The number of somatic cells (granulosa and theca cells) at each follicular stage was accounted for, and the updated flux was utilized to determine the metabolic behavior of the oocyte. For the somatic cells, flux balance analysis (FBA) was run by setting the objective function to maximize the pyruvate and biomass production subject to the experimental values (e.g., glucose intake, production of lactic acid, estrogen, hyaluronic acid). Except for primordial somatic cells, oxygen was restricted to be only consumed for somatic cells. Production of nitric oxide, carbon monoxide, L-alanine were included at all the somatic cell stages; sorbitol and fructose were added to the secondary to antral follicle objective functions, and hormone production (progesterone, testosterone and estrogen) and hyaluronic acid were included in the antral follicle objective function only. For the oocyte, the optimization function was composed of the biomass production and pyruvate consumption, subject to the oxygen consumption measured experimentally and consumption/production of lactic acid and consumption of sorbitol, fructose and ascorbic acid.

## Additional files

Additional file 1: Mice Recon 2, Matlab file

Additional file 2: Metabolites, reactions and enzymes present in Mice Recon 2. (XLSX 452 kb)

Additional file 3: Metabolic model functionalities. (XLSX 66 kb)

Additional file 4: OvoFol Recon 1, Matlab file

Additional file 5: Metabolites, reactions and enzymes present in OvoFol Recon 1. (XLSX 307 kb)

Additional file 6: Entrez ID, gene symbol, the community that the gene belongs to according to Infomap and the flow of information that is transferred through the enzymatic node. (XLSX 110 kb)

Additional file 7: Z-scores for each metabolic pathway using Infomap information for Mice Recon 2 and OvoFol 1. (XLSX 92 kb)

Additional file 8: Mean fold change and fdr-corrected *p*-values for each follicle cell type during ovarian follicle development. (XLSX 6750 kb)

Additional file 9: Supplementary Figures. (DOCX 13578 kb)

## Abbreviations

25aics: (S)-2-[5-Amino-1-(5-phospho-D-ribosyl)imidazole-4-carboxamido]succinate
2LS: Two layered secondary
5aizc: 5-amino-1-(5-phospho-D-ribosyl)imidazole-4-carboxylate
air: 5-amino-1-(5-phospho-D-ribosyl)imidazole
Akr1b3: Aldo-keto reductase family 1, member B3
Aldh1a1: Aldehyde dehydrogenase 1 family member A1
ANTRAL: Antral follicles
Atp1a1: ATPase Na+/K+, transporting subunit alpha 1
Bmp15: Bone morphogenetic protein 15
Ca_2_^+^: Calcium ion
CC: Cumulus cells
CcLA: Cumulus large antral
CcNSN: Cumulus granulosa cell in large antral follicles that present a non-surrounded oocyte nucleolus
CcSA: Cumulus small antral
CcSN: Cumulus granulosa cell in large antral follicles that present a surrounded oocyte nucleolus
CCTR: Cumulus granulosa cells between the non-surrounded to surrounded nucleolus
COBRA: Constraints-based reconstruction and analysis
ECM: Extracellular matrix
FBA: Flux-balance analysis
FC: Fold change
Fdas1: Fatty acid desaturase 1
fdr: False discovery rate
Fpgs: Folylpolyglutamate synthase
GEO: Gene Expression Omnibus
Hsd17b1: Hydroxysteroid 17-beta dehydrogenase 1
Hsd17b4: Hydroxysteroid 17-beta dehydrogenase 4
IACUC: Institutional Animal Care and Use Committee
IMP: International mouse phenotyping consortium
IVF: In vitro fertilization
IVM: In vitro maturation
LA: Large antral
LP: Linear programming
MLS: Multi-layer secondary
MR: Mural cells
MrLA: Mural large antral
MrSA: Mural small antral
Myo5b: Myosin Vb
NO: Nitric oxide
Nos1: Nitric oxide synthase
NSN: Non-surrounded oocyte nucleolus
OLA: Oocyte large antral
Ooep: Oocyte expressed protein
OPM: Oocyte primordial
OPR: Oocyte primary
OSA: Oocyte small antral
OSC: Oocyte secondary
Paics: Phosphoribosylaminoimidazole carboxylase and phosphoribosylaminoimidazolesuccinocarbox-amide synthase
PCOS: Polycystic ovarian syndrome
PINs: Protein-protein interaction networks
PMD: Primordial
PMSG: Pregnant mare serum gonadotropin
PND: Post-natal day
PR: Primary
PREANTRAL: Pre-antral follicles
RAB11A: Ras-related protein Rab-11A
SA: Small antral
SC: Secondary follicle
Scl3a2: Solute carrier family 3 member 2
SLA: Somatic large antral
Slc7a5: Solute carrier family 7 member 5
SN: Surrounded oocyte nucleolus
SPM: Somatic primordial
SPR: Somatic primary
SSA: Somatic small antral
SSC: Somatic secondary
Zp1: Zona pellucida glycoprotein 1
α-MEM: Alpha modified minimum essential medium
