# Supplementary material for: Dynamic genome-scale cell-specific metabolic models reveal novel inter-cellular and intra-cellular metabolic communications during ovarian follicle development
Source: BMC Bioinformatics. 2019 Jun 10;20:307. doi: 10.1186/s12859-019-2825-2 (PMC6558917; doi:10.1186/s12859-019-2825-2)
Supplement: Supplementary file 7 — Supplementary Figures. (DOCX 13578 kb) [file 12859_2019_2825_MOESM9_ESM.docx]

**Supplementary Figures**

**Figure S1. Comparison of metabolic pathways present in Mouse Recon 1 (green) with those in Mouse Recon 2 (purple).** A total of 12 metabolic pathways are new in the updated reconstruction.

**Figure S2. Effect of the somatic cell expansion during mouse ovarian follicle development.** A) Average intensity profiles of oocyte-specific genes in the oocyte microarray (in green) and in the follicle microarray (purple); B) Number of granulosa, theca, and total cells from isolated follicles as a function of follicle diameter; C) Effect of the granulosa cell expansion on the dilution of oocyte mRNA as a function of the follicle stage.


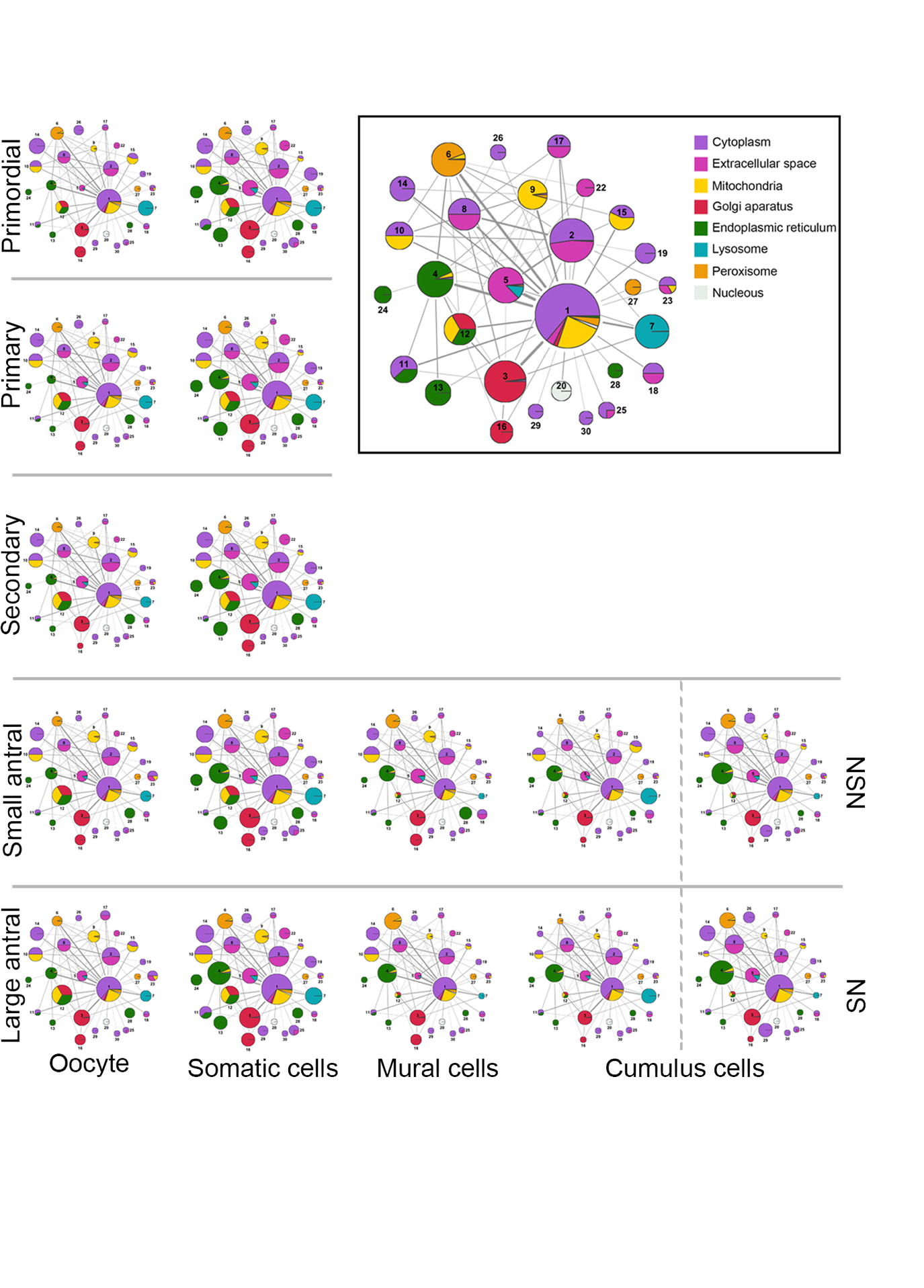


**Figure S3. Metabolic communities in the ovarian follicle based on flow between enzymes obtained from Infomap.** The sizes of the communities, defined as clusters of highly connected enzymes according to the flow of information that goes through the enzymatic nodes, and the width of the edges between communities are proportional to the information that flows through them. Communities are color-coded based on the proportion of enzymes that pertain to a given location normalized by the array intensity of each of the genes that encode enzymes present in the community. Therefore, larger communities have more active genes that encode for the enzymes present in that community.


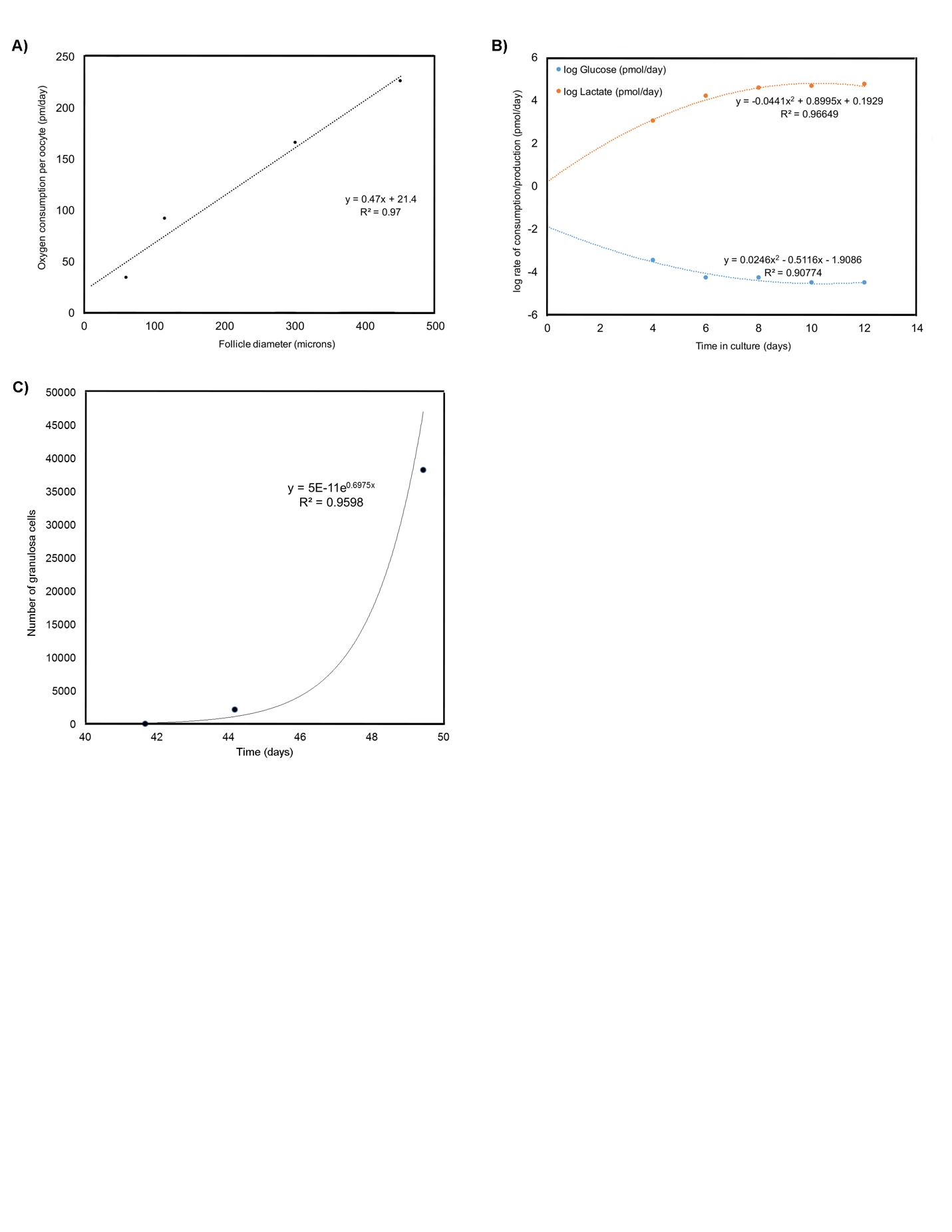


**Figure S4. Data to constrain context-specific follicle models.** A) Oxygen consumption as a function of the follicle size. Data obtained from Harris et al [1]; B) Lactose production and glucose consumption during *in vitro* follicle maturation. Modified from Harris et al. [2]; C) Granulosa cell growth rate during ovarian follicle development *in vivo*. Number of granulosa cells as a function of time. Data were fitted to an exponential growth function. Data obtained from Bristol-Gould et al. [3]

**Supplementary Tables**

**Table S1. Biological process gene ontologies of the new genes added to the latest Mouse Recon 1 from Human Recon 2*** Gene ontologies were obtained from GENEGO.

| Maps | Total | p-value | FDR | In Data |
| --- | --- | --- | --- | --- |
| [Oxidative phosphorylation](http://portal.genego.com/cgi/imagemap.cgi?id=920) | 105 | 2.8E-104 | 7.9E-102 | 79 |
| [Ubiquinone metabolism](http://portal.genego.com/cgi/imagemap.cgi?id=884) | 74 | 2.5E-48 | 3.5E-46 | 43 |
| [dCTP/dUTP metabolism](http://portal.genego.com/cgi/imagemap.cgi?id=874) | 75 | 9.3E-21 | 8.7E-19 | 25 |
| [dATP/dITP metabolism](http://portal.genego.com/cgi/imagemap.cgi?id=865) | 95 | 4.2E-19 | 3.0E-17 | 26 |
| [dGTP metabolism](http://portal.genego.com/cgi/imagemap.cgi?id=890) | 65 | 2.7E-17 | 1.5E-15 | 21 |
| [TTP metabolism](http://portal.genego.com/cgi/imagemap.cgi?id=872) | 66 | 1.5E-13 | 6.2E-12 | 18 |
| [Arachidonic acid production](http://portal.genego.com/cgi/imagemap.cgi?id=895) | 49 | 1.5E-13 | 6.2E-12 | 16 |
| [Linoleic acid / Rodent version](http://portal.genego.com/cgi/imagemap.cgi?id=2360) | 31 | 3.8E-10 | 1.4E-08 | 11 |
| [O-glycan biosynthesis / Human version](http://portal.genego.com/cgi/imagemap.cgi?id=2363) | 62 | 1.3E-09 | 4.0E-08 | 14 |
| [O-glycan biosynthesis](http://portal.genego.com/cgi/imagemap.cgi?id=914) | 60 | 8.7E-09 | 2.4E-07 | 13 |
| [Fatty Acid Omega Oxidation](http://portal.genego.com/cgi/imagemap.cgi?id=908) | 34 | 3.5E-06 | 9.0E-05 | 8 |
| [Cell cycle_Transition and termination of DNA replication](http://portal.genego.com/cgi/imagemap.cgi?id=707) | 28 | 9.5E-06 | 2.2E-04 | 7 |
| [ATP/ITP metabolism](http://portal.genego.com/cgi/imagemap.cgi?id=873) | 124 | 4.6E-05 | 9.9E-04 | 13 |
| [Unsaturated fatty acid biosynthesis](http://portal.genego.com/cgi/imagemap.cgi?id=830) | 62 | 5.5E-05 | 1.1E-03 | 9 |
| [CoA biosynthesis](http://portal.genego.com/cgi/imagemap.cgi?id=878) | 52 | 9.4E-05 | 1.8E-03 | 8 |
| [Retinol metabolism](http://portal.genego.com/cgi/imagemap.cgi?id=881) | 72 | 1.8E-04 | 3.2E-03 | 9 |
| [n-3 Polyunsaturated fatty acid biosynthesis](http://portal.genego.com/cgi/imagemap.cgi?id=900) | 63 | 3.7E-04 | 6.1E-03 | 8 |
| [Androstenedione and testosterone biosynthesis and metabolism p.2](http://portal.genego.com/cgi/imagemap.cgi?id=847) | 35 | 3.9E-04 | 6.1E-03 | 6 |
| [n-6 Polyunsaturated fatty acid biosynthesis](http://portal.genego.com/cgi/imagemap.cgi?id=899) | 64 | 4.1E-04 | 6.1E-03 | 8 |
| [DNA damage_Nucleotide excision repair](http://portal.genego.com/cgi/imagemap.cgi?id=3084) | 36 | 4.6E-04 | 6.2E-03 | 6 |
| [Androstenedione and testosterone biosynthesis and metabolism p.2/ Rodent version](http://portal.genego.com/cgi/imagemap.cgi?id=2326) | 36 | 4.6E-04 | 6.2E-03 | 6 |
| [Retinol metabolism / Rodent version](http://portal.genego.com/cgi/imagemap.cgi?id=2331) | 67 | 5.6E-04 | 7.2E-03 | 8 |
| [Heme metabolism](http://portal.genego.com/cgi/imagemap.cgi?id=880) | 103 | 6.4E-04 | 7.9E-03 | 10 |
| [Androstenedione and testosterone biosynthesis and metabolism p.1/ Rodent version](http://portal.genego.com/cgi/imagemap.cgi?id=2325) | 57 | 1.1E-03 | 1.2E-02 | 7 |
| [Mitochondrial unsaturated fatty acid beta-oxidation](http://portal.genego.com/cgi/imagemap.cgi?id=833) | 45 | 1.6E-03 | 1.8E-02 | 6 |
| [Cell cycle_Role of APC in cell cycle regulation](http://portal.genego.com/cgi/imagemap.cgi?id=472) | 32 | 1.9E-03 | 2.0E-02 | 5 |
| [Mitochondrial long chain fatty acid beta-oxidation](http://portal.genego.com/cgi/imagemap.cgi?id=832) | 83 | 2.3E-03 | 2.4E-02 | 8 |
| [Estradiol metabolism](http://portal.genego.com/cgi/imagemap.cgi?id=850) | 35 | 2.8E-03 | 2.7E-02 | 5 |
| [Estradiol metabolism / Human version](http://portal.genego.com/cgi/imagemap.cgi?id=2338) | 35 | 2.8E-03 | 2.7E-02 | 5 |
| [Estradiol metabolism / Rodent version](http://portal.genego.com/cgi/imagemap.cgi?id=2328) | 37 | 3.6E-03 | 3.4E-02 | 5 |
| [Glycolysis and gluconeogenesis p.3 / Human version](http://portal.genego.com/cgi/imagemap.cgi?id=2335) | 24 | 4.2E-03 | 3.7E-02 | 4 |
| [Glycolysis and gluconeogenesis p.3](http://portal.genego.com/cgi/imagemap.cgi?id=818) | 24 | 4.2E-03 | 3.7E-02 | 4 |
| [Nicotine metabolism in liver](http://portal.genego.com/cgi/imagemap.cgi?id=5932) | 56 | 4.8E-03 | 4.1E-02 | 6 |

* Gene ontologies were obtained from GENEGO.

**Table S2. Homologous genes present in Mouse Recon 2 that are not present in Human Recon 2**

| Gene Symbol | Gene Entrez | Protein GI | Protein Accession |
| --- | --- | --- | --- |
| BST1 | 683 | 168229159 | NP_004325.2 |
| CD36 | 948 | 48375178 | NP_000063.2 |
| AKR1B10 | 57016 | 223468663 | NP_064695.3 |
| FOLR2 | 2350 | 166064056 | NP_001107008.1 |
| GC | 2638 | 324021745 | NP_001191236.1 |
| LRP2 | 4036 | 126012573 | NP_004516.2 |
| LEPREL2 | 10536 | 28466983 | NP_055077.2 |
| LIG1 | 3978 | 4557719 | NP_000225.1 |
| LIG3 | 3980 | 73747829 | NP_039269.2 |
| RBP1 | 5947 | 195976807 | NP_002890.2 |
| RBP2 | 5948 | 40354214 | NP_004155.2 |
| RBP4 | 5950 | 55743122 | NP_006735.2 |
| SCARB1 | 949 | 132566680 | NP_001076428.1 |
| STRA6 | 64220 | 312261223 | NP_001185969.1 |
| CMPK2 | 129607 | 117606370 | NP_997198.2 |
| ABCG5 | 64240 | 11967969 | NP_071881.1 |
| SLC52A2 | 79581 | 13375682 | NP_078807.1 |
| LEPRE1 | 64175 | 186928835 | NP_071751.3 |
| PGP | 283871 | 108796653 | NP_001035830.1 |
| ABCG8 | 64241 | 11967971 | NP_071882.1 |
| NT5C3L | 115024 | 294459996 | NP_443167.4 |
| NADKD1 | 133686 | 146134341 | NP_001078880.1 |
| AK8 | 158067 | 22749187 | NP_689785.1 |
| GPX8 | 493869 | 192455698 | NP_001008398.2 |
| UGT2A3 | 79799 | 193211427 | NP_079019.3 |
| GADL1 | 339896 | 197383062 | NP_997242.2 |
| SLC6A19 | 340024 | 51468073 | NP_001003841.1 |
| NME5 | 8382 | 4505413 | NP_003542.1 |
| UGT2A1 | 10941 | 110611919 | NP_006789.2 |
| AKR1C3 | 8644 | 24497583 | NP_003730.4 |
| TECR | 9524 | 24475816 | NP_612510.1 |
| IDO2 | 169355 | 148539554 | NP_919270.2 |
| DHTKD1 | 55526 | 38788380 | NP_061176.3 |
| LEPREL1 | 55214 | 27764882 | NP_060662.2 |
| ITPK1 | 3705 | 217272844 | NP_001136065.1 |
| CCBL2 | 56267 | 56713254 | NP_001008661.1 |
| NT5C1A | 84618 | 14210538 | NP_115915.1 |
| NPC1L1 | 29881 | 156231351 | NP_037521.2 |
| LIG4 | 3981 | 148539894 | NP_001091738.1 |
| P4HA3 | 283208 | 33589818 | NP_878907.1 |
| UGT2A2 | 574537 | 333609245 | NP_001099147.2 |
| AKD1 | 221264 | 237858799 | NP_001138600.2 |
| UQCRHL | 440567 | 148229491 | NP_001083060.1 |

**Table S3. Analysis summary of ovarian follicle development microarrays**

| **Origin** | **Platform** | **Background correction** | **Normalization** | **Transformation** | **Detection** | **FC** | **Significance (p-value, fdr corrected)** |
| --- | --- | --- | --- | --- | --- | --- | --- |
| Oocyte  PMD to LA (GSE3351) | Affymetrix |  | quantile | log2 | Must be present in all repeats | 2.5 | 0.01 |
| Cumulus & mural cells  SA to LA  (GSE55845) | Affymetrix | rma | quantile | log2 | Must be present in all repeats, p-value<0.01 | 2 | 0.01 |
| Cumulus cells during the adquision of oocyte compentence,  NSN to SN (GSE36617) | Agilent | normexp | quantile |  | 4 out 5 repeats above 1.1 times the 95% intensity quantile of control probes | 1.5 | 0.01 |
| Somatic cells from entire follicle  PMD to LA | Illumina |  | rsn | vst | Must be present in all repeats, p-value<0.01 | 1.3 | 0.05 |

**Table S4. Estimations of metabolite production and consumption rates based on data from several literature resources**

|  |  | **PRIMORDIAL** | **PRIMARY** | **SECONDARY** | **SMALL ANTRAL** | **LARGE ANTRAL** |
| --- | --- | --- | --- | --- | --- | --- |
| **Follicle** | Diameter (µm)* | 18 | 60 | 115 | 300 | 450 |
|  | O2 (pmol/day)^†^ | -28.5 | -33.6 | -91.2 | -165.6 | -225.6 |
|  | Pyruvate (pmol/day)^‡^ | -0.7 | -19.7 | -24.5 | -40.3 | -40.1 |
|  | Glucose (pmol/day)^§^ | -0.3 | -79.4 | -3000.0 | -20000.0 | -35000.0 |
|  | L-lactate (pmol/day)^§^ | 1.4 | 1.5 | 1000.0 | 15000.0 | 51000.0 |
| **Oocyte** | O2 (pmol/day) | -28.5 | -33.6 | -91.2 | -165.6 | -225.6 |
|  | Pyruvate (pmol/day) | -0.7 | -19.7 | -24.5 | -40.3 | -40.1 |
| **Somatic cells** | Number of cells^‖^ | 14.8 | 411.1 | 2381.2 | 31705.9 | 94751.7 |
|  | O2 (pmol/day)** | -2.E+00 | -8.E-02 | -4.E-02 | -5.E-03 | -2.E-03 |
|  | Pyruvate (pmol/day) | -5.E-02 | -5.E-02 | -1.E-02 | -1.E-03 | -4.E-04 |
|  | Glucose (pmol/day) | -2.E-02 | -2.E-01 | -1.E+00 | -6.E-01 | -4.E-01 |
|  | L-lactate (pmol/day) | 9.E-02 | 4.E-03 | 4.E-01 | 5.E-01 | 5.E-01 |
|  | Progesterone (pmol/day)^¶^ |  |  | 3.E-05 | 4.E-05 | 4.E-05 |
|  | Estrogen(pmol/day)^¶^ |  |  | 2.E-04 | 2.E-04 | 2.E-04 |
|  | Testosterone (pmol/day)^¶^ |  |  | 3.E-04 | 4.E-04 | 4.E-04 |
|  | Haluronic acid (pmol/day) |  |  |  |  | 2.E-04 |
|  | Fructose (pmol/day) |  |  | -1.E-03 | -1.E-04 | -4.E-05 |
|  | Sorbitol (pmol/day) |  |  | -1.E-03 | -1.E-04 | -4.E-05 |

*Average size of collected follicles.

**Maximum oxygen consumption if the oocyte didn’t consume oxygen based on Fig. S4A [1]

^†^ Predicted from regression of Fig. S4A [1].

^‡^ Obtained from Harris et al [1].^§^ Predicted from regression Fig. S4B [2].

^‖^ Predicted from experimental measurements obtained from isolated follicles Fig S2A.

^¶^ Obtained from experimental measurements in *in vitro* grown follicles.

**Table S5. Differences in the metabolite composition of regular media used for *in vitro* follicle growth, plasma, and follicular fluid***

| Metabolite | Media | Plasma | Follicular fluid |
| --- | --- | --- | --- |
| gly | 1.2E-01 | 2.7E-02 | 5.3E-01 |
| ala_L | 6.8E-02 | 6.7E-02 | 7.5E-02 |
| alagln | 3.4E-01 | 0.0E+00 | 0.0E+00 |
| arg_L | 9.0E-02 | 1.1E-02 | 8.4E-02 |
| asn_L | 6.9E-02 | 4.0E-03 | 7.2E-02 |
| asp_L | 4.2E-02 | 1.2E-02 | 6.0E-01 |
| cys_L | 1.0E-01 | 1.0E-01 | 1.0E-01 |
| glc | 1.0E+00 | 1.0E+00 | 1.0E+00 |
| gln_L | 0.0E+00 | 7.7E-02 | 7.0E-02 |
| his_L | 3.6E-02 | 7.0E-03 | 8.4E-02 |
| ile_L | 7.2E-02 | 1.1E-02 | 7.1E-02 |
| leu_L | 7.2E-02 | 1.4E-02 | 1.4E-01 |
| lys_L | 7.2E-02 | 3.0E-03 | 1.0E-01 |
| met_L | 1.8E-02 | 6.0E-03 | 5.0E-02 |
| phe_L | 3.5E-02 | 8.0E-03 | 8.0E-02 |
| pro_L | 6.2E-02 | 6.2E-02 | 6.2E-02 |
| ser_L | 4.2E-02 | 1.3E-02 | 1.5E-01 |
| thr_L | 7.2E-02 | 3.9E-02 | 1.2E-01 |
| trp_L | 9.0E-03 | 8.0E-03 | 1.9E-02 |
| val_L | 7.1E-02 | 2.4E-02 | 1.3E-01 |
| ascb_L | 5.1E-02 | 5.1E-02 | 5.1E-02 |
| btn | 7.0E-05 | 7.0E-05 | 7.0E-05 |
| chol | 1.0E-03 | 1.0E-03 | 1.0E-03 |
| pnto_R | 4.0E-04 | 4.0E-04 | 4.0E-04 |
| fol | 4.0E-04 | 4.0E-04 | 4.0E-04 |
| ncam | 1.0E-03 | 1.0E-03 | 1.0E-03 |
| pydx | 9.0E-04 | 9.0E-04 | 9.0E-04 |
| ribflv | 5.0E-05 | 5.0E-05 | 5.0E-05 |
| thm | 5.0E-04 | 5.0E-04 | 5.0E-04 |
| aqcobal | 2.0E-04 | 2.0E-04 | 2.0E-04 |
| inost | 2.0E-03 | 2.0E-03 | 2.0E-03 |
| lipoate | 2.0E-04 | 2.0E-04 | 2.0E-04 |
| pyr | 1.8E-01 | 1.3E-01 | 8.2E-01 |
| ca2 | 3.3E-01 | 3.3E-01 | 3.3E-01 |
| co | 0.0E+00 | 0.0E+00 | 0.0E+00 |
| cl | 2.3E+01 | 2.3E+01 | 2.3E+01 |
| k | 9.6E-01 | 9.6E-01 | 9.6E-01 |
| na1 | 2.6E+01 | 2.6E+01 | 2.6E+01 |
| i | 3.2E-01 | 3.2E-01 | 3.2E-01 |
| o2 | 5.0E+01 | 5.0E+01 | 5.0E+01 |
| h2o | 1.0E+02 | 1.0E+02 | 1.0E+02 |
| co2 | 0.0E+00 | 0.0E+00 | 0.0E+00 |
| h | 5.0E+01 | 5.0E+01 | 5.0E+01 |
| hco3 | 4.8E+00 | 4.6E+00 | 4.8E+00 |
| pi | 1.9E-01 | 1.9E-01 | 1.9E-01 |
| so4 | 1.4E-01 | 1.4E-01 | 1.4E-01 |
| nh4 | 0.0E+00 | 0.0E+00 | 0.0E+00 |
| fe2 | 1.0E-01 | 1.0E-01 | 1.0E-01 |
| fe3 | 1.0E-01 | 1.0E-01 | 1.0E-01 |
| glyc | 0.0E+00 | 9.3E-03 | 0.0E+00 |
| hdca | 0.0E+00 | 3.6E-04 | 0.0E+00 |

**Table S5 (Continuation)**

| Metabolite | Media | Plasma | Follicular fluid |
| --- | --- | --- | --- |
| lnlc | 0.0E+00 | 0.0E+00 | 0.0E+00 |
| sel | 0.0E+00 | 0.0E+00 | 0.0E+00 |
| lac_L | 0.0E+00 | 4.1E-01 | 3.8E+01 |
| lac_D | 9.2E-02 | 0.0E+00 | 0.0E+00 |
| glu_L | 0.0E+00 | 2.8E-02 | 6.0E-01 |
| tyr_L | 3.6E-02 | 1.0E-01 | 6.5E-02 |
| taur | 0.0E+00 | 1.1E-01 | 2.1E+00 |
| Lcystin | 1.1E-01 | 2.0E-02 | 0.0E+00 |
| Ex_octa | 0.0E+00 | 1.5E-03 | 0.0E+00 |
| adrn | 0.0E+00 | 9.3E-05 | 0.0E+00 |
| crvnc | 0.0E+00 | 9.3E-03 | 0.0E+00 |
| dhdascb | 0.0E+00 | 3.7E-04 | 0.0E+00 |
| ksi | 0.0E+00 | 2.6E-05 | 0.0E+00 |
| ptrc | 0.0E+00 | 1.9E-05 | 0.0E+00 |
| thymd | 0.0E+00 | 3.7E-05 | 0.0E+00 |
| prpp | 0.0E+00 | 9.3E-04 | 0.0E+00 |
| gam | 0.0E+00 | 5.4E-05 | 0.0E+00 |
| but | 0.0E+00 | 1.9E-04 | 0.0E+00 |
| glygly | 0.0E+00 | 9.3E-05 | 0.0E+00 |
| nrpphr | 0.0E+00 | 3.0E-07 | 0.0E+00 |
| pe_hs | 0.0E+00 | 1.0E-06 | 0.0E+00 |
| CE5853 | 0.0E+00 | 0.0E+00 | 0.0E+00 |
| CE1926 | 0.0E+00 | 0.0E+00 | 0.0E+00 |
| idp | 0.0E+00 | 0.0E+00 | 0.0E+00 |
| sbt_D | 0.0E+00 | 2.4E-03 | 0.0E+00 |
| sucr | 0.0E+00 | 3.3E-04 | 0.0E+00 |
| man | 0.0E+00 | 7.4E-03 | 0.0E+00 |
| 2hb | 0.0E+00 | 9.1E-02 | 0.0E+00 |
| dhap | 0.0E+00 | 2.4E-03 | 0.0E+00 |
| dgsn | 0.0E+00 | 1.9E-04 | 0.0E+00 |
| udpg | 0.0E+00 | 2.9E-02 | 0.0E+00 |
| ksi | 0.0E+00 | 2.6E-05 | 0.0E+00 |
| ptrc | 0.0E+00 | 1.9E-05 | 0.0E+00 |
| thymd | 0.0E+00 | 3.7E-05 | 0.0E+00 |
| prpp | 0.0E+00 | 9.3E-04 | 0.0E+00 |
| gam | 0.0E+00 | 5.4E-05 | 0.0E+00 |
| but | 0.0E+00 | 1.9E-04 | 0.0E+00 |
| glygly | 0.0E+00 | 9.3E-05 | 0.0E+00 |
| nrpphr | 0.0E+00 | 3.0E-07 | 0.0E+00 |
| pe_hs | 0.0E+00 | 1.0E-06 | 0.0E+00 |
| CE5853 | 0.0E+00 | 0.0E+00 | 0.0E+00 |
| CE1926 | 0.0E+00 | 0.0E+00 | 0.0E+00 |
| idp | 0.0E+00 | 0.0E+00 | 0.0E+00 |
| sbt_D | 0.0E+00 | 2.4E-03 | 0.0E+00 |
| sucr | 0.0E+00 | 3.3E-04 | 0.0E+00 |
| man | 0.0E+00 | 7.4E-03 | 0.0E+00 |
| 2hb | 0.0E+00 | 9.1E-02 | 0.0E+00 |
| dhap | 0.0E+00 | 2.4E-03 | 0.0E+00 |
| dgsn | 0.0E+00 | 1.9E-04 | 0.0E+00 |
| udpg | 0.0E+00 | 2.9E-02 | 0.0E+00 |

* Data obtained from Harris et al [4] and the human metabolomics database [5].

**SUPPLEMENTARY NOTE 1**

**DEVELOPMENT OF MOUSE RECON 2**

Mouse Recon 2 was developed based on the latest human metabolic model, Recon 2.03 [6], and we employed a similar methodology to that applied for the first mouse reconstruction [7], which was based on Human Recon 1 [8].

**Model reconstruction**

The reconstruction was performed through a series of iterative steps, with the goal of reducing ambiguities when merging the two reconstructions:

1. Metabolite name consistency was improved using the CHEBI [9] and Metacyc [10] (<http://www.metacyc.org/>) version 17.0 databases. Metabolite names and synonyms, formulas, charges, InChi strings, and Smiles strings collected from the two databases were compared for each metabolite present in the Human Recon 2 model and in the most updated Mouse Recon 1 model [11], giving priority to the CHEBI database. CHEBI identification numbers and InChi fields, if available from CHEBI or Metacyc databases, were added to the human and mouse reconstructions if any one of the following conditions were satisfied: a) exact match between the InChi fields in the model and in the CHEBI database; or b) exact match between the metabolite (charged) chemical formula, charge, and name with the corresponding counterparts in the CHEBI database (for the metabolite name, synonyms were included). By guaranteeing only one match between the database and the metabolite, the likelihood that the metabolite has any isomers was reduced.
2. The abbreviation format of the mouse metabolites was changed to match the human abbreviation format.
3. Each metabolite that pertains to any Human Recon 2 reaction not present in the Mouse Recon 1 model was contrasted against the Mouse Recon 1 metabolites to identify their mouse counterpart. These were the elements checked in order of priority: InChi strings, metabolite abbreviations, cellular location (i.e., cytoplasm, mitochondria), chemical formula and charge (this avoided duplication of unique metabolites that are present in different cellular compartments), and metabolite abbreviation. During the comparison, several mismatches were identified and corrected through an iterative process.
4. Several reactions were updated in the human and/or mouse metabolic models: a) the glucose transport was improved by the addition of the glucose/sodium co-transporter, SLC5A1, other non-sodium dependent glucose transporters and collagen production. All of these were added to Human Recon 2 before updating the Mouse Recon 1 model; b) several reactions for ascorbic acid metabolism were upgraded in the mouse model as it differs substantially from the human ascorbic acid metabolism; c) some reactions from androgen and estrogen metabolism were included in the mouse model.
5. Reactions present in Human Recon 2, but not present in Mouse Recon 1, were included in Mouse Recon 1 in two steps. In the first step, we added the reactions involving genes that are not included in Mouse Recon 1. For each reaction added into the model, several precautions were taken to avoid adding duplicated metabolites or reactions (i.e., due to an unbalanced reaction). In the second step, all the reactions that were not included in Mouse Recon 1 yet have a human homologue and are part of Human Recon 2 were adjoined as well, maintaining the same precautions with respect to duplications. Homologous genes in human and mouse were obtained from HomoloGene (<https://www.ncbi.nlm.nih.gov/homologene)> build 67 downloaded as of 08/16/2013.
6. Mass balance and charge balance was checked and identified for multiple reactions for which either the metabolite charge and chemical formula was missing, was incorrect, or the number of atoms for each component was not balanced. Each imbalanced reaction was manually checked and corrected when possible. As a consequence, this procedure was iterated again from step 3 to step 6 until no correctable reactions were detected. The mouse model has 154 non-balanced reactions, all of them included in the Human Recon 2 model, except the *CytOxe-* and *SOProd* reactions.

The MATLAB Mouse Recon 2 model is in **File S1**, and the model reactions, metabolites, and genes are summarized in **File S2**.

**SUPPLEMENTARY NOTE 2: GAP FILLING AND MODEL FUNCTIONALITY VALIDATION**

**Gap filling**

Blocked reactions and dead end metabolites (reactions whose non-secreted metabolites are not connected to other parts of the metabolic networks) were identified with FASTCC, an algorithm that identifies the consistent part of a constraint metabolic model [12]. The curated reactions from the KEGG database (<http://www.genome.jp/kegg/)> were employed to identify possible reactions that could be added to the Mouse Recon 2 model to unblock some of the non-flux reactions and decrease the number of dead end metabolites using FASTCORE [12] by giving priority to core metabolites reactions (weight 100) versus transport reactions between the cellular compartments or exchange reactions (weight 10). A total of 626 reactions could potentially be added to Mouse Recon 2 to close some of the gaps, yet a cross-reference of the mouse reactions in the KEGG database yielded a list of 288 that occur in mice. We excluded those that did not occur between metabolites already present in the Mouse Recon 2 model. With the same criteria, we added several exchange reactions. No transport reaction was added at this time. Taken together, a total of 133 new reactions and 86 genes associated with them were added to Mouse Recon 2.

**Model functionality**

Subsequently, the new Mouse Recon 2 was examined against several functions (**File S3**), such as production of biomass, production of pyruvate and lactate under anaerobic conditions from glucose, pyruvate consumption, hormone production (estrogen, androgen, testosterone, and progesterone), and fructose and sorbitol production.

**SUPPLEMENTARY NOTE 3: SOMATIC CELL TRANCRIPTOME**

The somatic cell specific transcriptional abundance was derived from the ovarian follicle transcriptional abundance after subtracting oocyte-specific genes. The basis for this assumption was the relative low abundance of oocyte-specific genes encoding for enzymes or transporters within the ovarian follicle transcriptional abundance. For instance, oocyte-specific (e.g., *Bmp15*, *Zp1*, *Ooep*) gene expression was observed to decrease from the secondary to the antral follicle stages (**Fig. S2A**), which resulted from the mitotic expansion of the granulosa cells after the secondary stage (**Fig. S2B**). This expansion diluted the oocyte gene expression in the stage-specific microarrays (**Fig. S2C**). Transcripts in the ovarian follicle transcriptome that followed that unique temporal pattern, and that were also changing their expression in the oocyte transcriptomic data (FC≥2 and p-value (fdr-corrected) ≤ 0.01) represent 6% of the ovarian follicle transcriptome. Of these genes, only 1% of these genes encoded enzymes or transporters from the oocyte. Thus, the somatic cell transcriptional abundance was determined by the ovarian follicle transcriptome less the oocyte specific genes. The likelihood that a gene present in somatic cells may have been removed was expected to be substantially less than 1%.

**SUPPLEMENTARY NOTE 4: DEVELOPMENT OF CONTEXT-SPECIFIC METABOLIC MODELS**

Metabolic networks for the oocyte and somatic cells during ovarian follicle development (primordial, primary, secondary and antral) were extracted using the FASTCORE algorithm [13]. Secreted metabolites at each follicle stage were obtained from the context-specific models, constructed as previously described, and constrained with experimental parameters when they were available: a) biomass rate of production of somatic cells were obtained based on the total cell number (**Fig. S2A**), and the growth rates of follicle pools (**Fig. S2B**) [3]. The rate of production of biomass agrees with the exponential growth observed before [14]; b) fluxes for estradiol and progesterone were estimated from cultures of oocyte-cumulus cell complexes [15] and assuming double the flux of testosterone to estradiol; c) experimental measurements from hyaluronic acid (HA) [16]; d) sorbitol and fructose fluxes were estimated as 10% of the pyruvate production, as it has been speculated that they are produced by the somatic cells and consumed by the oocyte [17]; e) oxygen and pyruvate consumptions by the oocyte [1]; f) glucose consumption and lactate production [2]; and g) normalized ratios of plasma concentrations [4] to glucose. Data are summarized in **Table S4**.

Total metabolite flow uptake was determined iteratively, so that the final estimated pyruvate flux coincided with the experimentally measured pyruvate production by the somatic cells. Metabolites produced or consumed by the somatic cells were added to or subtracted from the initial flux to the somatic cells. The number of somatic cells at each follicular stage was accounted for, and the updated flux was utilized to determine the metabolic behavior of the oocyte. For the somatic cells, FBA was run setting the objective function to maximize the pyruvate and biomass production subject to the experimental values. Except for primordial somatic cells, oxygen was restricted to be only consumed. Production of sorbitol and fructose were added to the secondary and antral follicle objective function and hormone production (progesterone, testosterone, and estrogen) to the antral follicle objective function only. For the oocyte, the optimization function was composed of the biomass production and pyruvate consumption, subject to the oxygen consumption measured experimentally.

**SUPPLEMENTARY REFERENCES**

1. Harris SE, Leese HJ, Gosden RG, Picton HM: **Pyruvate and oxygen consumption throughout the growth and development of murine oocytes**. *Molecular reproduction and development* 2009, **76**(3):231-238.

2. Harris SE, Adriaens I, Leese HJ, Gosden RG, Picton HM: **Carbohydrate metabolism by murine ovarian follicles and oocytes grown in vitro**. *Reproduction* 2007, **134**(3):415-424.

3. Bristol-Gould SK, Kreeger PK, Selkirk CG, Kilen SM, Mayo KE, Shea LD, Woodruff TK: **Fate of the initial follicle pool: empirical and mathematical evidence supporting its sufficiency for adult fertility**. *Dev Biol* 2006, **298**(1):149-154.

4. Harris SE, Gopichandran N, Picton HM, Leese HJ, Orsi NM: **Nutrient concentrations in murine follicular fluid and the female reproductive tract**. *Theriogenology* 2005, **64**(4):992-1006.

5. Wishart DS, Jewison T, Guo AC, Wilson M, Knox C, Liu YF, Djoumbou Y, Mandal R, Aziat F, Dong E *et al*: **HMDB 3.0-The Human Metabolome Database in 2013**. *Nucleic Acids Res* 2013, **41**(D1):D801-D807.

6. Haraldsdottir HS, Thiele I, Fleming RMT: **Comparative evaluation of open source software for mapping between metabolite identifiers in metabolic network reconstructions: application to Recon 2**. *J Cheminformatics* 2014, **6**.

7. Sigurdsson MI, Jamshidi N, Steingrimsson E, Thiele I, Palsson BO: **A detailed genome-wide reconstruction of mouse metabolism based on human Recon 1**. *Bmc Syst Biol* 2010, **4**.

8. Duarte NC, Becker SA, Jamshidi N, Thiele I, Mo ML, Vo TD, Srivas R, Palsson BO: **Global reconstruction of the human metabolic network based on genomic and bibliomic data**. *Proceedings of the National Academy of Sciences of the United States of America* 2007, **104**(6):1777-1782.

9. Degtyarenko K, De Matos P, Ennis M, Hastings J, Zbinden M, McNaught A, Alcantara R, Darsow M, Guedj M, Ashburner M: **ChEBI: a database and ontology for chemical entities of biological interest**. *Nucleic Acids Res* 2008, **36**:D344-D350.

10. Caspi R, Altman T, Dreher K, Fulcher CA, Subhraveti P, Keseler IM, Kothari A, Krummenacker M, Latendresse M, Mueller LA *et al*: **The MetaCyc database of metabolic pathways and enzymes and the BioCyc collection of pathway/genome databases**. *Nucleic Acids Res* 2012, **40**(D1):D742-D753.

11. Heinken A, Sahoo S, Fleming RM, Thiele I: **Systems-level characterization of a host-microbe metabolic symbiosis in the mammalian gut**. *Gut Microbes* 2013, **4**(1):28-40.

12. Vlassis N, Pacheco MP, Sauter T: **Fast reconstruction of compact context-specific metabolic network models**. *PLoS Comput Biol* 2014, **10**(1):e1003424.

13. Vlassis N, Pacheco MP, Sauter T: **Fast Reconstruction of Compact Context-Specific Metabolic Network Models**. *Plos Comput Biol* 2014, **10**(1).

14. Pedersen T: **Determination of follicle growth rate in the ovary of the immature mouse**. *Journal of reproduction and fertility* 1970, **21**(1):81-93.

15. Vanderhyden BC, Tonary AM: **Differential regulation of progesterone and estradiol production by mouse cumulus and mural granulosa cells by A factor(s) secreted by the oocyte**. *Biol Reprod* 1995, **53**(6):1243-1250.

16. Salustri A, Yanagishita M, Underhill CB, Laurent TC, Hascall VC: **Localization and synthesis of hyaluronic acid in the cumulus cells and mural granulosa cells of the preovulatory follicle**. *Dev Biol* 1992, **151**(2):541-551.

17. Sutton-McDowall ML, Gilchrist RB, Thompson JG: **The pivotal role of glucose metabolism in determining oocyte developmental competence**. *Reproduction* 2010, **139**(4):685-695.
